# Digital Response Test in Epilepsy assesses interictal epileptiform discharge effects in real time

**DOI:** 10.1111/epi.18629

**Published:** 2025-10-03

**Authors:** Andreas von Allmen, Diyuan Lu, Caroline Jagella, Yasmina Abukhadra, Rune Markhus, Jochen Triesch, Margaret Gopaul, Lawrence J. Hirsch, Felix Rosenow, Hal Blumenfeld, Heinz Krestel

**Affiliations:** ^1^ Department of Neurology Yale School of Medicine New Haven Connecticut USA; ^2^ Frankfurt Institute for Advanced Studies Frankfurt am Main Germany; ^3^ Department of Neuroscience Yale School of Medicine New Haven Connecticut USA; ^4^ National Centre for Epilepsy, full member of European Reference Network on Rare and Complex Epilepsies EpiCARE, Division of Clinical Neuroscience Oslo University Hospital Oslo Norway; ^5^ Department of Neurology West Haven Veterans Affairs Hospital, West Haven, Connecticut, USA; ^6^ Department of Neurology, Epilepsy Center Frankfurt Rhine‐Main, University Medical Center Frankfurt and Center for Personalized Translational Epilepsy Research (CePTR) Goethe University Frankfurt am Main Germany; ^7^ Department of Neurosurgery Yale School of Medicine New Haven Connecticut USA; ^8^ Present address: Helmholtz Institute Munich Germany; ^9^ Present address: Department of Computer Science Stanford University Stanford California USA

**Keywords:** driving safety, EEG, interictal epileptiform discharge effect, machine learning, transitory cognitive impairment

## Abstract

**Objective:**

Interictal epileptiform discharges (IEDs) in people with epilepsy (PWE) can impair cognitive functions and increase reaction time (RT) and the likelihood of missed reactions. These effects are not routinely assessed, because reliable methods for detecting IEDs of variable appearance in real time and suitable tests to measure IED effects do not yet exist. The objective was to assess different IED effects using new artificial intelligence and medical electronics.

**Methods:**

The Digital Response Test in Epilepsy (DigRTEpi) consisted of a laptop and electronic circuits in a closed loop. Our model with Markov Transition Fields and a deep neural network (ResNet34) visualized the electroencephalogram (EEG) and classified the resulting images. IED detection triggered stimuli in a driving game or in a new cognitive assessment, the interictal Automated Responsiveness Test (iART). DigRTEpi was validated in a prospective case series with 20 people with focal and generalized epilepsies. During offline analysis, sensitivity, specificity, false‐positive IED detection rate, latency of EEG classification, IED‐induced RT prolongation, virtual crashes, and impaired responses to neuropsychological tasks were determined.

**Results:**

The model detected IEDs with 84% sensitivity and 96% specificity in our training dataset. In the prospective study with 20 PWE, median sensitivity was 90% (95% confidence interval [CI] = .81–.95), and false‐positive IED detection rate was 2.8 (95% CI 2.1–5.9). The ongoing EEG was classified window‐by‐window in a median 98.7 ms (95% CI = 98.0–99.4). Median RT prolongation and crash probability due to IEDs were 43.8 ms (95% CI = 20.3–64.7) and .9% (95% CI = 0–6.0) per person, respectively. Two patients (10%) had delays of >100 ms, found to be clinically relevant in our prior publication. IEDs caused four patients (20%) each to respond incorrectly or miss answers to neuropsychological tasks. The median false‐positive IED detection rates were 2.8/min (95% CI = 2.1–5.9; driving game) and 2.1/min (95% CI = 1.5–3.2; iART).

**Significance:**

By effectively detecting IEDs of variable morphology in real time, DigRTEpi assessed the severity of IED‐associated transitory impairment of virtual driving and cognition to improve personalized care.

**Key points**

- The Digital Response Test in Epilepsy (DigRTEpi) provides time‐locked analysis of interictal epileptiform discharge (IED) effects.
- A supervised machine learning model detects IEDs with variable appearance in real time and triggers patient tasks.
- A simple driving game with obstacles to avoid measures IED effects on reaction time and crashes and can be used to assess driving skills.
- Videos of the interictal Automated Responsiveness Test (iART), presenting neuropsychological tasks, examine IED impact on cognition.
- DigRTEpi provides laboratory measurements of IED effects with real‐world applicability and can be used for all types of epilepsy.

## 1 INTRODUCTION

People with epilepsy (PWE) are affected not only by seizures but also by short electric changes in the electroencephalogram (EEG) that occur between seizures, known as interictal epileptiform discharges (IEDs). IEDs can have effects on behavior and cognition that range from negligible impairment to the transient inability to perform an action. Although IEDs are much shorter in duration compared to seizures, ranging from half a second to a few seconds, if they occur in series or runs, they can be up to 2000 times more frequent than seizures in temporal lobe epilepsy.^1^ We and others have found that IEDs can prolong reaction time (RT) and cause missed reactions, and that these clinical correlates are associated with electrophysiological IED‐features such as spatial extent of the electrical field, morphology, duration, and amplitude.^2^, ^3^, ^4^, ^5^ We showed that a simple driving game is sufficient to measure IED‐associated effects on RT and virtual accidents (crashes) with good sensitivity and specificity, and that IED effects are comparable in younger and older PWE.^2^ By describing the cumulative rate of missed reactions as a nonlinear function of RT prolongation due to IEDs, a risk for missed reactions can be predicted (what we called the predictive power of IEDs); for example, the risk is 20% at a mean RT prolongation of 100 ms and rises to 50% at an average RT prolongation of 150 ms.^2^ There are experimental tests to assess IED‐induced deficits, but there are no standardized tests in practice to detect IED‐associated effects on daily social functioning, because of a lack of automatic, objective, fast, and versatile methods. In addition, there is currently no consensus in the scientific literature about the ideal test to measure IED effects in relation to driving ability.^6^

Earlier devices for automatically detecting IEDs and measuring their effects typically used amplitude threshold detectors to differentiate IEDs from normal EEG and triggered a simple visual or auditory stimulus to which the patients were asked to react. Sensitivity and specificity of IED detection were variable, and only patients with a certain type of epilepsy or a certain type of IED, that is, homogeneous patient groups, could be tested on an experimental basis.^3^, ^7^, ^8^, ^9^ With manually triggered stimuli, the sensitivity and specificity of IED detection were higher on average, but the stimuli often occurred at the end of an IED, so that the maximum IED effect could not be measured.^2^ Finally, the current criteria of identifying solitary IEDs were derived from a narrow, carefully assessed population of people with focal epilepsy and are unlikely to account for the significant inter‐ and intraindividual variability of IEDs, or to be applied to abnormal waveforms when the necessary IED criteria are not met.^10^ The variable morphology of IEDs, their different clinical correlates, the compromise between test accuracy and speed of IED detection, and the limited ability to assess the impact of IEDs on various brain functions have made real‐time measurement of IED effects difficult in practice. This study focused on the ultrafast identification of IED series with variable morphology and the comprehensive assessment of their maximum effect by using IEDs to trigger different tests and measure patient responses. As established neuropsychological tests are not timed to detect IED‐associated transitory cognitive impairment (TCI),^e.g., 11^ we newly developed the interictal Automated Responsiveness Test (iART).^12^

The Digital Response Test in Epilepsy (DigRTEpi) was tested in a prospective proof‐of‐concept study with 20 PWE to accurately detect IED series with variable morphology in real time in generalized and focal epilepsies, measure the clinical correlates of IEDs, and assess their relevance as minor or clinically relevant for the individuals tested.

## 2 MATERIALS AND METHODS

### 2.1 The EEGs used for training, optimization, and testing the IED‐detection model

A total of 125 deidentified scalp EEGs of 66 patients (median age = 24 years, 34 females) with different types of epilepsy (28 focal, 38 generalized onset) were obtained from three university hospitals with the permission of the ethics committees of Bern University Hospital and University of Bern, Switzerland (KEK No. 165/10, Basec PB_2017_00574), the Epilepsy Center Frankfurt Rhine‐Main, Department of Neurology, University Medical Center and Goethe University Frankfurt, Germany (2022‐841), and the Oslo National Epilepsy Centre, Norway (REK sør‐øst 2019/647). Table S1 provides information about the EEGs and patients.

Eighty‐two EEGs from 53 patients were from Bern. Forty‐four EEGs were simultaneously recorded with a simple reaction test to single flashes.^2^ Thirty‐eight EEGs were simultaneously recorded with a simple driving game.^2^ Three long‐term scalp EEGs from one patient who underwent presurgical epilepsy evaluation were from Frankfurt. Forty EEGs from 12 patients were from Oslo. Of these, 12 EEGs were recorded with a flash test and a driving game, whereas 11 were recorded with a cognitive test and five with a realistic driving simulator (Appendix S1). EEGs were included in supervised machine learning when they contained at least one IED. A total of 6187 IEDs in 125 EEGs with a total duration of almost 47 h (average duration = 22 min/EEG) were labeled by hand (Appendix S1, p. 2). IED prevalence was 2.2/min. The IED distribution was 1910:2652:1625 for Bern:Frankfurt:Oslo. Interrater reliability was repeatedly checked. It was high and mostly related to discussions about when an IED begins and ends. We did not systematically calculate a kappa statistic, because the aim here was not only to detect single spike–waves and polyspike–waves, but also to detect serial IEDs and, above all, to determine the effects of these EEG changes.

### 2.2 IED definition

IEDs were defined essentially as previously described.^2^ On a clinical level, they were “not recognizable” by regular observation. On an electrophysiological level, they were defined as a series of epileptiform potentials (>1 potential, also termed IED‐bursts hereafter) without evolution in amplitude and frequency (<1 Hz over the duration of the discharge). Each IED‐burst was classified as focal or generalized according to the width of its electrical field. A generalized IED‐burst was classified as “typical” if it consisted of classically configured spike–waves and/or polyspike–waves with reasonably constant organization and amplitude throughout (“well organized”). Generalized atypical IED‐bursts consisted of more bluntly configured spike–waves and sharp theta activity and were less well organized, often with variable amplitude over time (“other type”). Focal IED‐bursts were defined independently of their morphology.

For supervised learning of the model, IED‐bursts were hand‐labeled as soon as they had a minimum duration of 400 ms (half of the window used for conversions of microvolt transitions from datapoint to datapoint). In principle, there was no upper limit to the duration, except perhaps 10 s to exclude possible electrographic seizures. An IED with a duration of 400 ms (including aftergoing wave) was usually a single IED.^13^, ^14^, ^15^ These were absolutely in the minority (estimated << 5%) and were subsumed under the term IED‐burst in this article.

### 2.3 Digital Response Test in Epilepsy (DigRTEpi)

**FIGURE 1:**
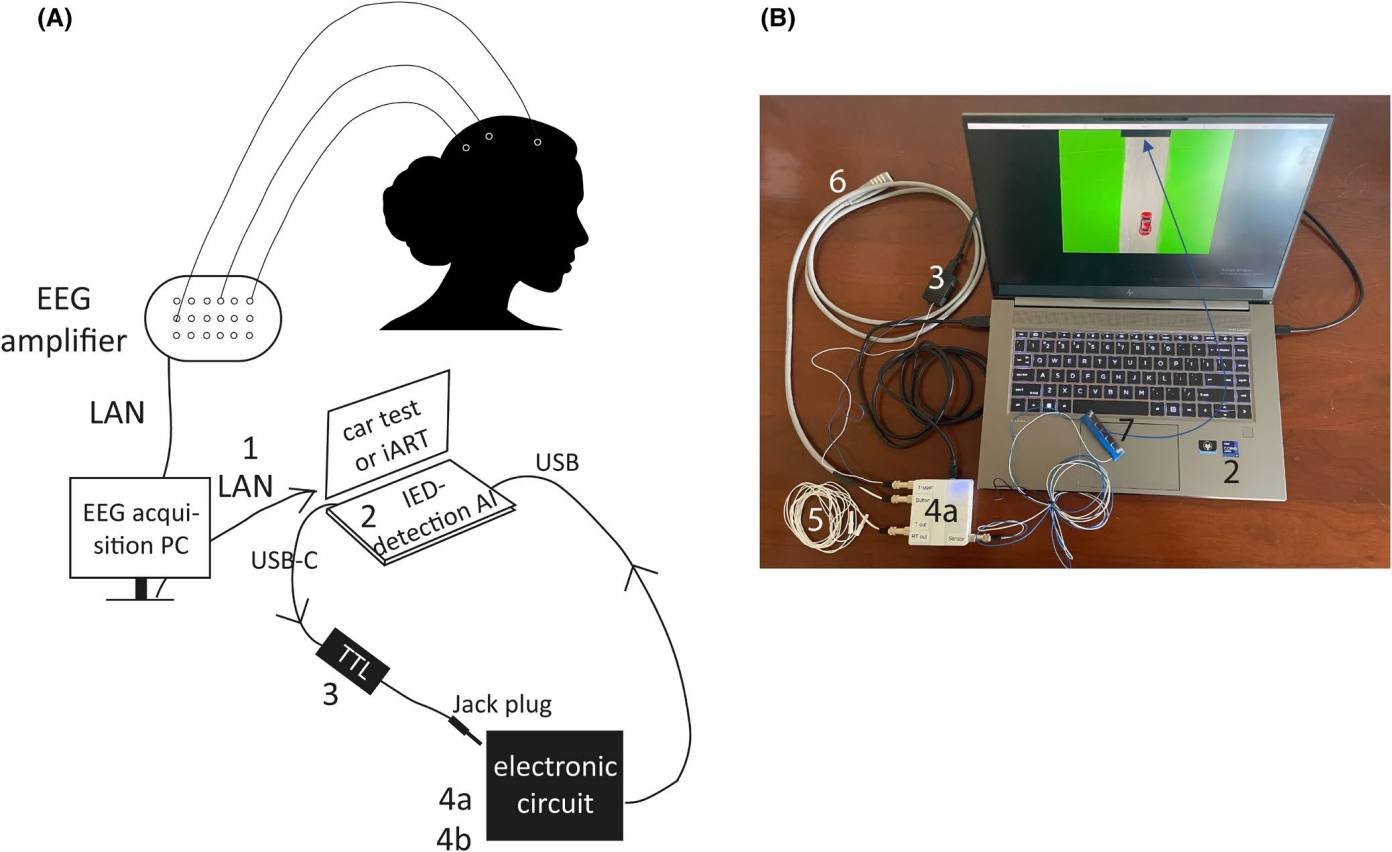
Architecture of the Digital Response Test in Epilepsy. (A) Voltage signals were continuously recorded in an EEG using scalp electrodes (symbolized by small white circles on the woman's head) and transmitted via cable to an EEG‐amplifier, where they were converted into digital values with a certain number of bits using an analog–digital converter. The signals were amplified and transmitted via a local area network (LAN) cable to an EEG‐acquisition personal computer (PC), where the EEG was displayed and recorded. A TCP/IP protocol written for EEG acquisition stations from Micromed and Natus continuously transmitted the EEG in packets to the study laptop (2) using a second LAN cable (1). Once the study laptop received enough microvolt values from the ongoing EEG recording, the model processed the data at once. When an interictal epileptiform discharge (IED) burst was classified, the transistor–transistor logic (TTL) chip (3) gated the electronic circuits (4), which in turn controlled the tests and recorded the patients' responses. Electronic circuit 4a controlled the car test (shown on the monitor in panel B). Circuit 4b controlled the interictal Automated Responsiveness Test (iART) used to assess IED effects on cognition. (B) (2) Study laptop; (3) TTL chip; (4a) electronic circuit for the car test; (5) jack to two‐pin connector used to indicate the activation of electronic circuit 4a as a square‐wave signal in an additional channel of the EEG recording; (6) push‐button for stopping the stopwatch in the car test's electronic circuit, thereby ending the reaction time (RT) measurement; (7) photovoltaic sensor for detecting the appearance of an obstacle on the road, which starts the stopwatch in the car test's electronic circuit and begins the RT measurement. The use of a photovoltaic sensor eliminated the digital latency of the closed loop that occurred between the car test's electronic circuit and the appearance of the obstacle on the laptop screen. The digital latency was unpredictable and had to be measured for each IED detection, that is, for each activation of the closed loop. The blue arrow indicates where the photovoltaic sensor was attached to the laptop screen above a black horizontal bar.

DigRTEpi consisted of a study laptop that ran our IED‐detection model and the patient tests, as well as two external devices in a closed loop that converted IED detection into trigger signals, controlled the tests, and recorded patient responses (Figure 1; detailed description in Appendix S1, p. 2).

### 2.4 IED‐detection model

**FIGURE 2:**
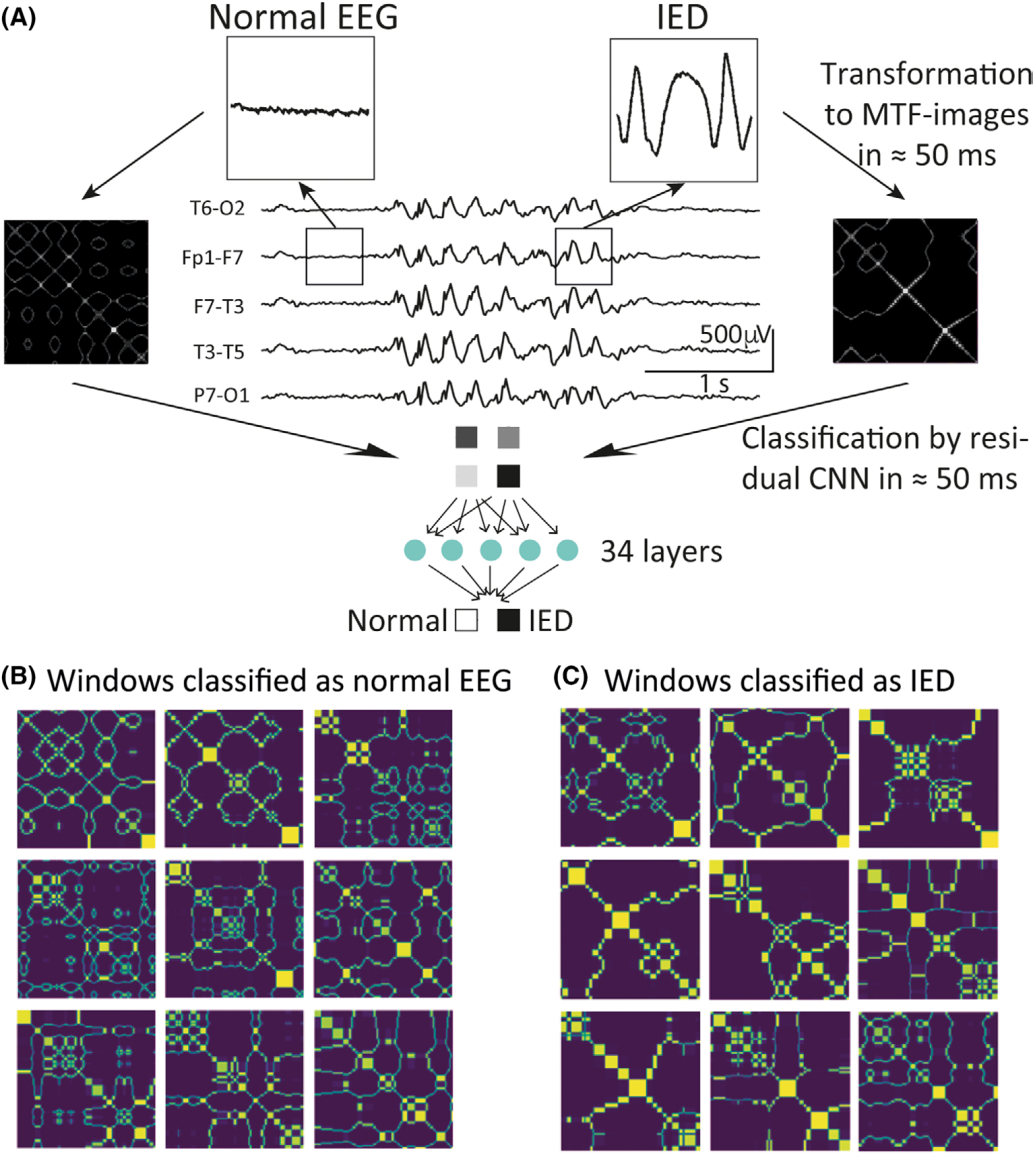
How the interictal epileptiform discharge (IED) detection model works. (A) Each sliding window (box) contained 200 μV values of the ongoing electroencephalographic (EEG) recording (at 256‐Hz sampling frequency) that were transformed into images using Markov Transition Fields (MTFs) and classified with a residual convolutional neural network (CNN) with 34 layers called ResNet34. (B, C) Colored MTF image examples of normal EEG (B) and IED‐bursts (C).

The IED‐detection model converted an ongoing EEG recording portion‐by‐portion into images using an advanced visualization technique called Markov Transition Fields (MTFs) and classified the images using a deep convolutional neural network (CNN; Figure 2, detailed description in Appendix S1, p. 8). The sliding‐window technique was used to portion the EEG in windows containing 200 μV values. One window had a duration of 781 ms at 256 Hz sampling frequency. The window moved forward at a step size of 195 ms (50 μV values). Windows thus overlapped by ≈600 ms (150 μV values). The model took one window each to discretize its 200 μV values into 32 quantiles based on their amplitude, calculate their transition probabilities between the quantiles, normalize, and present them in an *Markov Transition matrix* (first matrix). These transition probabilities were extracted for all consecutive pairs of microvolt values of a window in an *MTF‐matrix* (second matrix) and visualized. Each image represented the amplitude dynamics of an EEG segment and was classified by a residual CNN with 34 layers (ResNet34).

IED‐detection model optimization mainly focused on modifying ResNet performance. Experiments were conducted with variable MTF parameters, but ultimately, the default settings were used, except that we utilized grayscale image arrays resulting from the transformation of the EEG‐windows instead of color to reduce computational effort (detailed description in Appendix S1, p. 11). The optimization of ResNet34 involved addressing class imbalance and applying transfer learning and fine‐tuning. Briefly, class imbalance, that is, the bias of many more datapoints of normal EEG than IED‐bursts on classifier performance, was tackled by replacing the global cross entropy (CE) loss function, which comes by default with ResNet34, with the focal balanced CE loss function. The focal balanced CE loss function balanced the uneven amount of datapoints during normal EEG and IED‐bursts and focused the training of the model on data that were difficult to learn.^16^ ResNet34 was trained with various artificial ratios of datapoints of normal EEG and IED‐bursts. Transfer learning aimed to improve classification performance by pretraining ResNet34's neural layer weights with naturalistic images using the global CE loss function.^17^ The global CE loss function was then replaced with the focal CE loss function, and ResNet34 was retrained using EEGs. This modified model was additionally fine‐tuned, that is, it was trained a third time with a subset of EEGs with good signal‐to‐noise ratio. The fully optimized model consisted of pretrained ResNet34 with the focal balanced CE loss function, training with IED‐bursts and normal EEG in a 1:5 ratio, and fine‐tuning with a subset of IEDs with good signal‐to‐noise ratio. The results of IED‐detection model optimization are described in Appendix S1, p. 19, and Table S3.

### 2.5 Driving game and ultrarapid cognitive assessment (iART)

The simplified driving game (referred to hereafter as car test for reasons of consistency with previous publications^2^, ^3^, ^4^) measured RTs and crashes in response to an obstacle (Video S1, Appendix S1, p. 5). The car could not swerve. Patients had 1 s to change lane by button‐press; otherwise, the car crashed into the obstacle. The RT prolongation and crash probability were calculated using the RTs and crashes during IED‐bursts and normal EEG from a test session. The IED‐associated crash probability was the number of IED‐associated crashes divided by the number of all IED‐bursts in a patient's test session, multiplied by 100 (%). The crash probability due to IED‐bursts was the IED‐associated crash probability adjusted for the percentage of crashes during normal EEG in one session. The average value per person corresponded to the crash probability measured in this study over the mean test session duration of approximately 20 min. The cumulative risk of a crash was calculated using the session RT prolongation due to IED‐bursts and a nonlinear relationship between these two parameters. The average cumulative crash risk per person was the calculated cumulative crash probability that would be expected in general for a given patient. It should serve as a guide if no crashes were measured in a short test session (detailed car test study parameters are provided in Appendix S1, p. 16).

**FIGURE 3:**
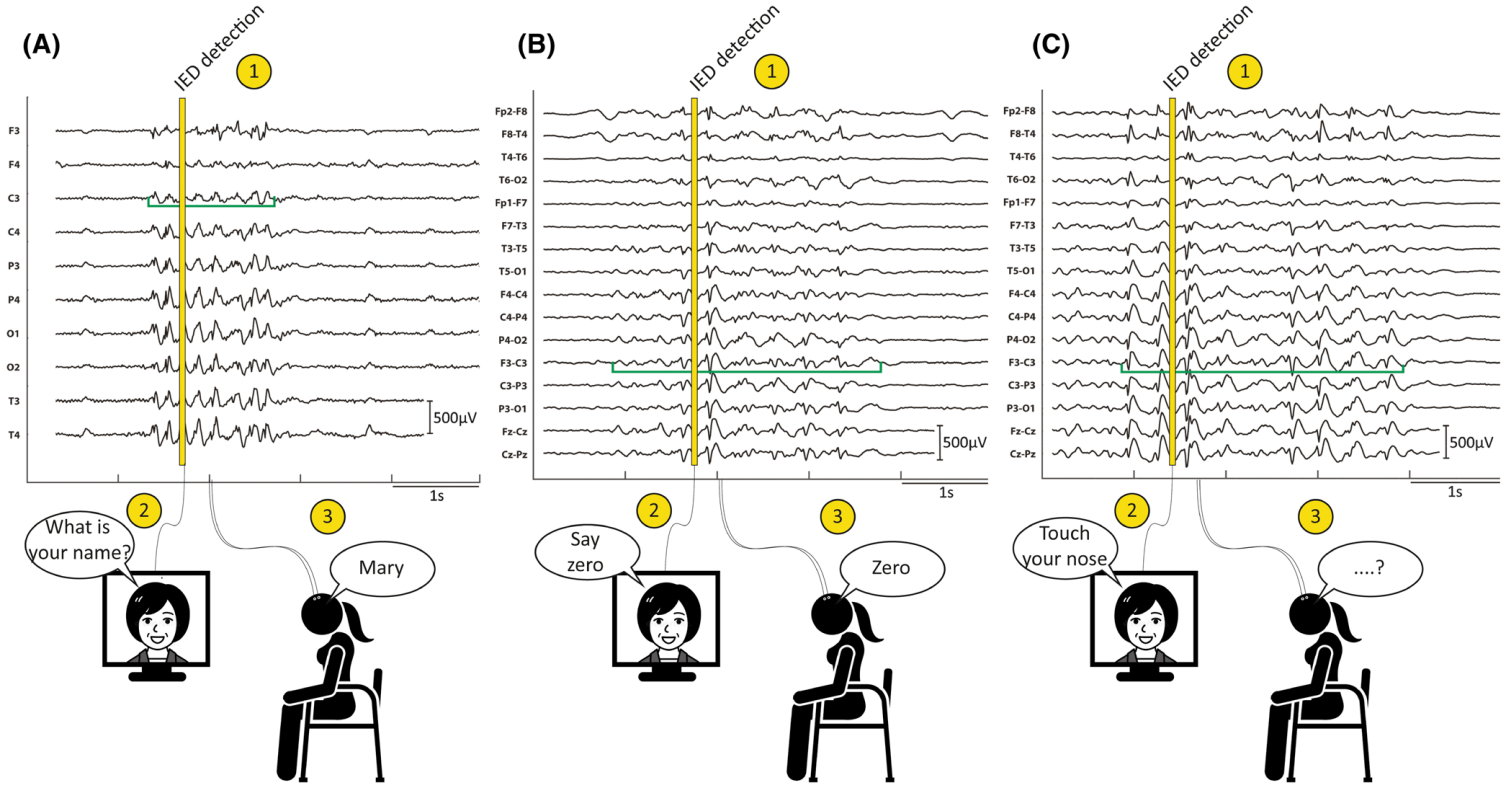
How the interictal Automated Responsiveness Test (iART) works. iART assessed the impact of interictal epileptiform discharge (IED) bursts on cognition by playing one of 40 videos with neuropsychological tasks contemporaneously with an IED‐burst. To increase the sensitivity of detecting transitory cognitive impairment due to IED‐bursts, the videos can be played in 17 different languages to test people with epilepsy in their native language. In each panel, the green open‐topped bracket indicates the beginning, duration, and end of each IED‐burst, and the numbers 1–3 indicate the sequence of events. (A, B) Patients responded correctly during shorter IED‐bursts. (C) A longer IED‐burst over 3 s was associated with a missed response.

iART measured correct, incorrect, or missing responses to varied neuropsychological tasks (Figure 3; Appendix S1 p. 6; Table S4).^12^ Because the IED‐bursts triggered the brief videos, the neuropsychological tasks were contemporaneously presented with the IED‐bursts and their effects, that is, TCI could be assessed. The model not only triggered tasks when it detected IED‐bursts (true‐positive classification), but also when it mistook EEG changes during normal EEG for IED‐bursts (false‐positive classification). We used the patient's responses to the stimuli triggered by these false‐positive classifications as baseline values for the IED‐burst effects in both the car test and iART. Therefore, each patient served as their own control (detailed iART study parameters are provided in Appendix S1, p. 18).

### 2.6 Prospective pilot study

Fifteen PWE from the Epilepsy Center Frankfurt Rhine‐Main and five PWE from the Yale Comprehensive Epilepsy Center with IED‐bursts in earlier EEGs were included in the prospective study regardless of medication. Patients had focal (including unifocal and multifocal disorders) and generalized epilepsies of genetic, structural, or unknown cause (13 focal, seven generalized onset; the detailed medical history including epilepsy type is described in Table S2). The only exclusion criterion was a prominent cognitive deficit. The study complied with the Declaration of Helsinki and the local ethics committees (Frankfurt 2022‐841, Yale IRB protocol ID 2000034300). All patients, or their legal representatives, gave informed consent/assent.

### 2.7 Statistical analysis

See Appendix S1, Methods, p. 18.

## 3 RESULTS

**FIGURE 4:**
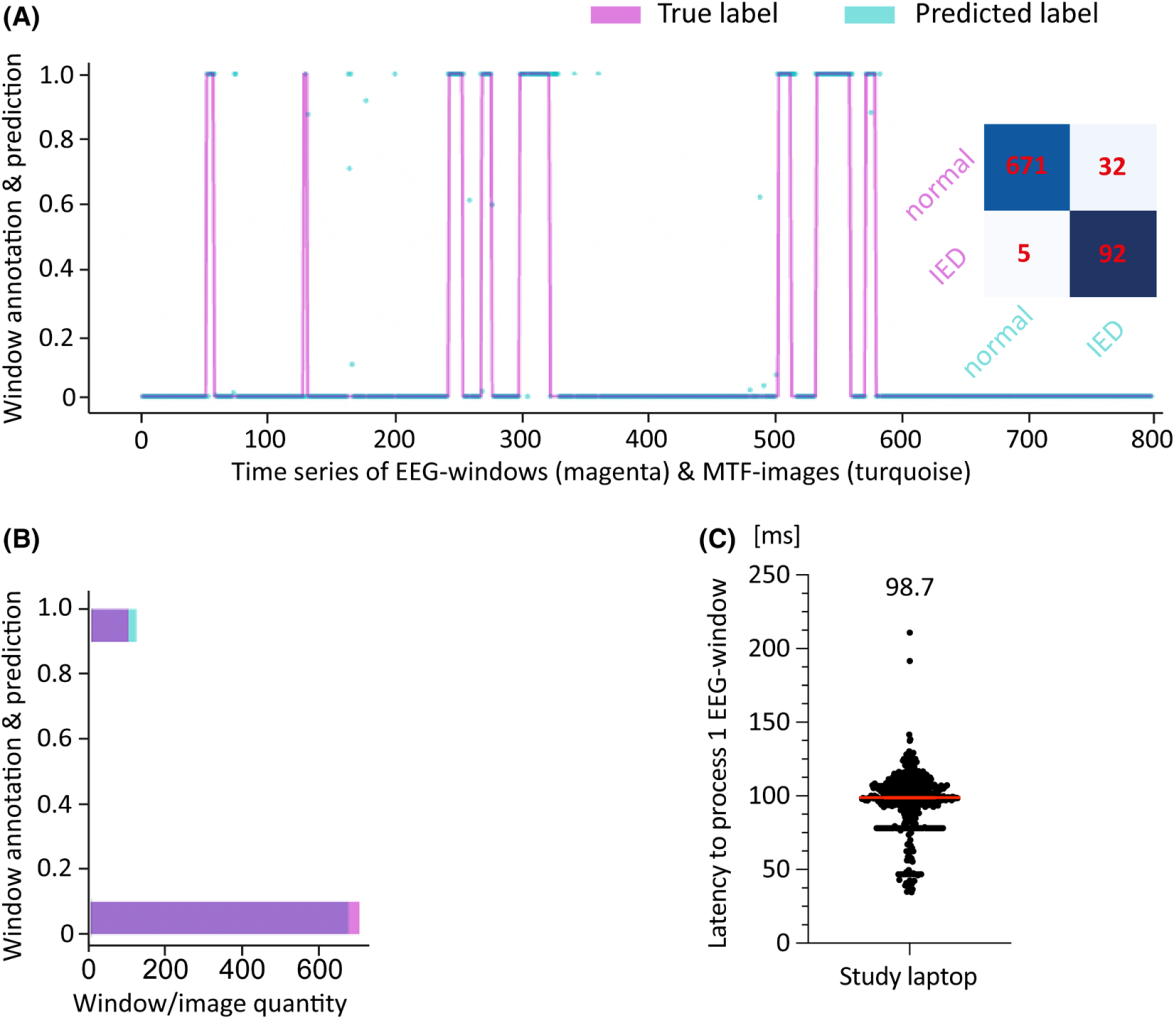
Performance of the interictal epileptiform discharge (IED) detection model. (A) Classification of the test segment of an individual electroencephalogram (EEG) from a patient with focal epilepsy of unknown cause (Bern Patient 137_SC, Table S1) who took the car test (with eyes open). The x‐axis shows the EEG as a time series of windows (in magenta, with points of the line representing overlapping windows) and Markov Transition Field (MTF) images (turquoise dots). Each window contained 200 μV values (at 256 Hz sampling frequency) that were associated with hand‐labeled annotations and thus represent the “true label.” The MTF‐images were generated from the windows by the model and classified. They represent the “predicted label” (turquoise dots). Because each window or image had a duration of ≈800 ms and overlapped with the next window or image by ≈600 ms, the number of windows or images on the x‐axis of this logistic diagram represented the time. The x‐axis has units between 0 and 800, corresponding to a duration of 156 s. The test segment comprised 15% of this EEG and lasted 195 s, so only part of the test segment of this routine EEG is shown. The y‐axis of the logistic diagram shows both the window annotation and the prediction probability, ranging between [0] and [1]. When the EEG was segmented during preprocessing, all microvolt values of hand‐labeled IED‐bursts obtained the label [1]. All microvolt values outside the hand‐labeled IED‐bursts obtained the label [0]. When most microvolt values (≥150) of a window carried the label [1], it was defined as an IED‐burst, otherwise as normal EEG. Thus, the magenta baseline and spikes represent windows of normal EEG and IED‐bursts. The model assigned each MTF‐image a prediction probability. When the prediction probability was ≥.99, the image was classified as an IED‐burst. When the turquoise dots overlaid the magenta spikes at [1] or at [0], they were correctly classified by the model. The turquoise dots with a prediction probability of ≥.99, which did not overlap with the magenta peaks, included the 32 MTF‐images that represented normal EEG, but were false‐positively classified as IED‐bursts (inset, confusion matrix). Ninety‐two MTF images were correctly classified as IED‐bursts (confusion matrix). They were distributed over the eight magenta peaks in the logistic diagram, indicating that multiple MTF‐images corresponded to an IED‐burst in the EEG. (B) The number of MTF‐images and the number of EEG‐windows are overlaid in a horizontal bar diagram for quantification. The bar at [1] is approximately seven times shorter than the bar at [0], indicating the unbalanced class ratio between IED‐bursts and normal EEG that was characteristic of this EEG. The part of the bar at [1] that is only colored in turquoise but not in purple (color mix between turquoise and magenta) represents the number of false‐positive classifications. (C) Average latency of the IED‐detection model. Each of the 522 points in this scatter plot represents the latency required to process (i.e., transform and classify) a window. The points were taken randomly from three different EEG recordings. The median latency in milliseconds is given above the scatter plot and is shown as a red horizontal bar together with the 95% confidence interval. As the three red horizontal lines of the statistics are so close together, they appear as one line. The “horizontal black bar” at approximately 75 ms (latency) is not a statistic, but individual datapoints printed side by side by the GraphPad Prism statistics program that overlap.

Classification performance of the fully optimized model was examined using concatenated (i.e., connected) test segments of the EEG training dataset (Appendix S1, p. 19, Table S3), and using individual EEGs (Bern, Figure 4A; Frankfurt, Figure S2A; Oslo, Figure S2B). For the individual EEG from Bern, sensitivity was .96, specificity was .99, F1‐score was .96, Matthews correlation coefficient was .91, negative predictive value was .99, area under the receiver operating characteristic curve was .99, and area under the precision–recall curve was .96 (for metrics see Appendix S1, p. 19). The accuracy was 96.9% when the EEG classification results of the three individual EEGs from Bern, Frankfurt, and Oslo were combined (Table S2, columns BO–BW). The fully optimized IED‐detection model took a median of 98.7 ms (95% confidence interval [CI] = 98.0–99.4 ms; Figure 4C) and a mean of 94.3 ms (SD = 21.6; data not shown) to transform and classify an EEG window using the graphics processing unit of the study laptop (NVIDIA GeForce RTX 3060 with 6 GB RAM). IED detection activated the closed‐loop circuit. The digital latencies (with a median value of 68 ms) from activation of the closed loop to the appearance of an obstacle in the car test (because RTs were measured in millisecond resolution) are described in Appendix S1, p. 22 and Table S2, column AE.

**FIGURE 5:**
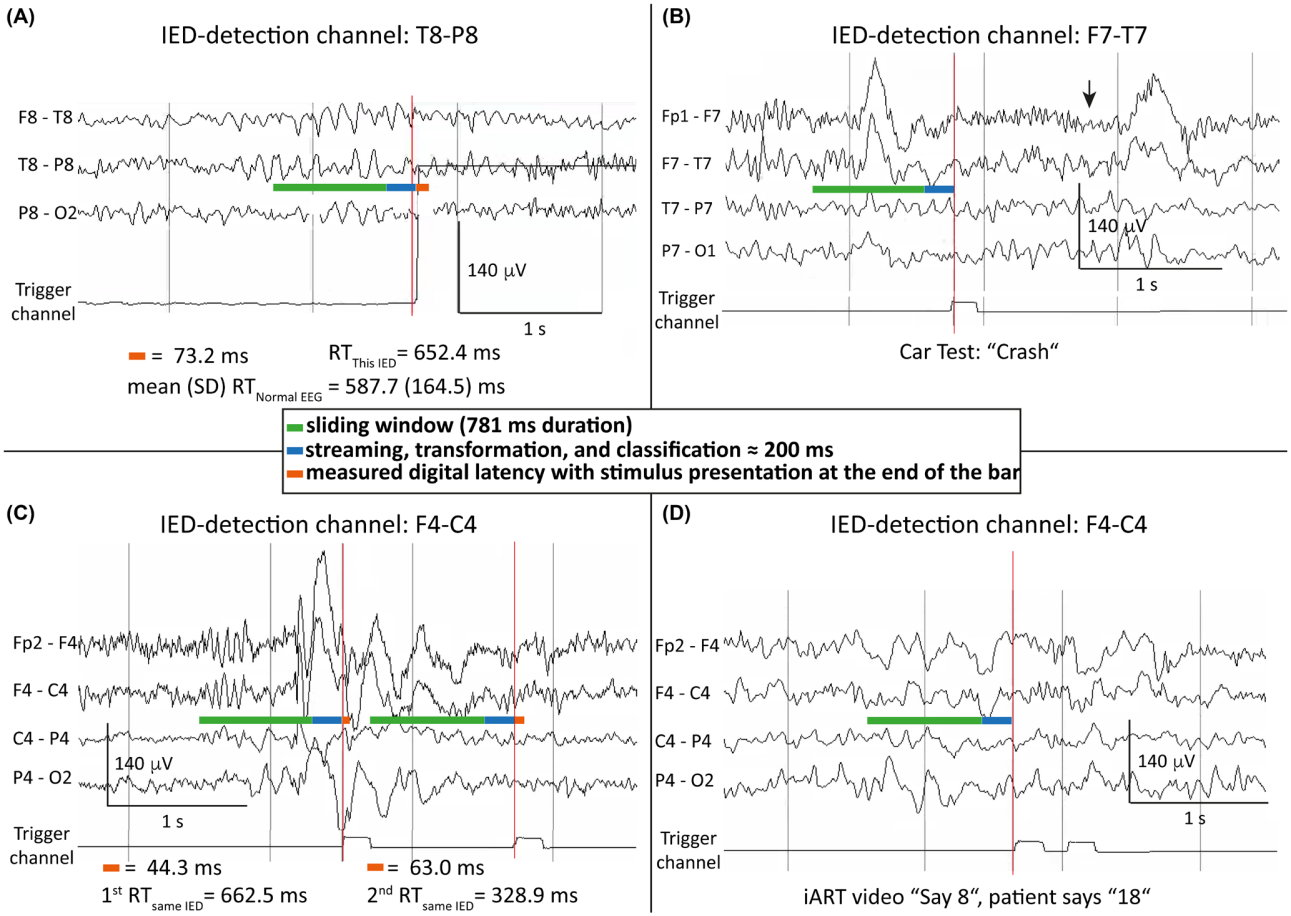
Variable interictal epileptiform discharge (IED) bursts are automatically detected, a stimulus is triggered, and their effects are measured. The model used a single EEG‐channel in bipolar montage for IED‐burst detection, written at the top of each panel. The box in the middle applies to all panels. The green horizontal bar indicates the duration of the sliding window. The blue horizontal bar indicates the duration of the system for transmitting and processing the EEG datapoints of a window (100 ms for streaming + 100 ms for transformation and classification). The orange horizontal bar indicates the recorded digital latency of the closed‐loop circuit for reaction time (RT) measurement in the car test only. The visual stimulus (obstacle in the car test, video in interictal Automated Responsiveness Test [iART]) appeared on the laptop monitor when the digital latency ended. The trigger channel was a connection between the electronic circuit controlling the car test or iART and the EEG‐amplifier and indicated the activation of the electronic circuit with an upstroke of a square‐wave signal. The trigger was manually highlighted by a vertical red line in the EEG‐reader software in each panel. (A) Patient 16 with focal epilepsy of unknown origin and focal theta sharp activity in the EEG (Table 1, Table S2). The digital latency between triggering and on‐screen appearance of the obstacle was 73.2 ms. The RT during this IED burst was 652 ms, compared to the mean RT during normal EEG of the entire session of 588 ms (SD ≈ 165 ms). (B) Patient 17 with idiopathic generalized epilepsy and atypical generalized IED‐bursts (Table 1, Table S2). The discharge occurred between the two high‐amplitude delta waves and lasted approximately 3 s. The digital latency was not recorded for crashes, because only RTs were measured in millisecond resolution. The patient failed to react within 1 s, and the car crashed into the obstacle (arrow). (C) Adolescent Patient 20 with atypical absence epilepsy (Table 1, Table S2). During generalized spike–wave activity of approximately 2.5‐s duration, an obstacle was triggered twice. The first trigger's digital latency was 44.3 ms, and the RT was 662.5 ms. The second trigger's digital latency was 63.0 ms, and the RT was 328.9 ms. (D) Adolescent Patient 20 with atypical absence epilepsy (Table 1, Table S2). An episode of generalized sharp(ish) theta–delta activity of approximately 2.5‐s duration triggered a video in iART, asking the patient to “Say 8.” The patient responded incorrectly with “18.” During the normal EEG of this test session, there were no incorrect or missing responses that would indicate inattentiveness.

DigRTEpi was assessed in two arms of a nonsignificant risk study with 20 PWE to collect proof‐of‐concept data. In the car test, IED‐bursts increased median session RT by 43.8 ms (minimum = 18.3, maximum = 135.1; *p* < .01, Wilcoxon test; Table 1, column 6). Median crash probability due to IED‐bursts was .9% (95% CI = 0–6; minimum = −2.9, maximum = 27.5; *p* < .01, Wilcoxon test; Table 1, column 13). Figure 5 shows examples of how the model detected IED‐bursts of variable appearance and measured RT prolongations and crashes in the car test, as well as incorrect responses in iART. Because IED‐associated crashes were rarely measured in a 20‐min test session, the cumulative crash risk was calculated. The per‐person cumulative crash risk was a median 5.7% (95% CI = 1.9–10.7; Table 1, column 14) and correlated well with the measured crash probability due to IED‐bursts (*R* ^2^ = .7, *p* < .01; correlation between columns 13 and 14 in Table 1).

**TABLE 1:** Test results of the prospective pilot study: Using the car test.

1	2	3	4	5	6	7	8	9	10	11	12	13	14	15	16	17
Patient	Test duration, min	Triggers, *n*	IEDs, *n*	Mean RT during normal EEG, ms	ΔRT due to IEDs, ms	Duration of IEDs without crash, ms	Crashes due to IEDs, *n*	Duration of IEDs with crash, ms	Crashes during normal EEG, *n*	Measured IED‐associated crash probability, %	Measured crash probability during normal EEG, %	Measured crash probability due to IED ‐bursts, %	Calculated cumulative crash risk, %	IED detection sensitivity	IED detection specificity	False‐positive IED‐ detection rate/min
1	26	73	44	428.0	60.7	1853	4	2892	1	10.0	3.4	6.6	8.0	.86	.99	2.8
2	8	78	49	356.1	78.4	1709	1	3194	0	2.0	0	2.0	10.7	.90	.99	2.1
3	12	178	72	476.9	18.3	1358	0	716	0	.0	0	.0	.8	.89	.99	2.3
4	14	61	29	627.6	39.2	671	1	1307	1	3.4	3.2	.2	2.9	.95	.99	2.6
5	16	135	26	480.8	20.3	859	0		0	.0	0	.0	.9	.83	.97	5.9
6	36	246	51	474.2	56.5	1210	2	12 367	0	3.9	0	3.9	6.7	.91	.99	2.8
7	21	123	17	547.9	135.1	1697	5	3313	2	29.4	1.9	27.5	43.1	.81	.99	2.8
8	23	77	0	621.3											.98	3.5
9	26	12	76	554.5	10.4	880	1	1775	1	1.3	.8	.5	.4	.67	.99	.5
10	20	51	1	574.0	32.3	1140	0		0	.0	0	.0	1.9	1.00	.99	1.8
11	28	425	97	564.7	48.3	1201	1	1061	3	1.0	.9	.1	4.6	.97	.94	9.7
12	9	205	112	593.2	59.7	2125	13	2293	5	11.6	5.6	6.0	7.7	.57	.99	9.3
13	14	305	104	747.4	4.7	1265	1	1373	1	1.0	.5	.5	.2	.99	.95	14.0
14	21	111	45	612.9	64.7	1809	2	1339	2	4.4	3.1	1.3	9.3	.95	.99	2.7
15	30	65	1	566.1	36.9	664	0		0	.0	0	.0	2.6	1.00	.99	2.1
16	21	53	15	587.7	20.0	1842	8	2185	14	53.3	38.9	14.4	14.4	.79	.99	1.7
17	18	31	5	504.9	71.4	741	1	1488	1	16.6	4.5	12.1	12.1	.60	.99	1.3
18	9	49	11	607.1	29.5	903	0		1	0	2.9	−2.9	2.5	.83	.98	3.6
19																
20	35	343	93	481.1	105.1	1434	6	1505	2	6.5	.8	5.7	27.4	.91	.97	6.9
Mean	20.4	137.9	44.6	547.3	49.5	1297.8	2.6	2629.1	1.9	8.0	3.7	4.3	8.7	.86	.98	4.2
SD	8.4	113.3	36.8	87.1	32.9	445.4	3.4	2808.1	3.2	13.2	8.7	7.1	10.6	.13	.01	3.5
Count	19	19	19	18	18	18	18	14	18	18	18	18	18	18	18	19
Median	21.0	78.0	44.0	565.4	43.8	1237.5	1.0	1640.0	1.0	2.7	.9	.9	5.7	.90	.99	2.8
Median (95% CI)					43.8 (20.3–64.7)	1238 (880–1709)		1640 (1307–3194)		2.7 (0–10.0)	.9 (0–3.2)	.9 (0–6.0)	5.7 (1.9–10.7)	.90 (.81–.95)	.99 (.98–.99)	2.8 (2.1–5.9)

*Note*: Column 1: The tested patients whose medical history is described in Table S2. Column 2: The duration of the session with the car test in minutes. Column 3: The total number of triggers generated by the car test's electronic circuit based on the IED‐burst detection by the model and identified as true‐positive and false‐positive triggers in the offline analysis. Column 4: The number of IED‐bursts detected by the model that were visually confirmed as true‐positive triggers in the offline analysis. Column 5: Mean RT during normal EEG on a session level. The patients' reactions to false‐positive triggers generated by the model during normal EEG were taken as RTs during normal EEG in the offline analysis. Column 6: RT prolongation due to IED‐bursts (ΔRT due to IEDs), calculated from the mean RT during IED‐bursts minus the mean RT during normal EEG on a session level. Column 7: The mean duration of all IED‐bursts without crashes from one session. Column 8: The total number of crashes during IED‐bursts in a test session. Column 9: The mean duration of all IED‐bursts without crashes from one session. Column 10: The total number of crashes during normal EEG in a test session. Column 11: The measured IED‐associated crash probability is the number of crashes during IED‐bursts in relation to all IED‐bursts in one session, given as a percentage. Column 12: The measured crash probability during normal EEG is the number of crashes during normal EEG in relation to all triggers by the model during normal EEG in one session, given as a percentage. Column 13: The measured crash probability due to IED‐bursts is the IED‐associated crash probability adjusted for the percentage of crashes during normal EEG in one session (i.e., subtracted from each other). Column 14: The calculated cumulative crash risk was predicted from the RT prolongation due to IED‐bursts for each test session using a nonlinear equation described in Appendix S1 and is what would be expected in general for a given patient. It should serve as a guide if no crashes were measured in a short test session. Column 15: Sensitivity of the model to detect IED‐bursts in the EEGs recorded together with the car test. Column 16: Specificity of the model to detect IED‐bursts in the EEGs recorded together with the car test. Column 17: False‐positive IED‐detection rate in the car test, calculated as the number of false‐positive triggers (by the model during normal EEG) divided by the duration of the test session in minutes.

Abbreviations: CI, confidence interval; SD, standard deviation; EEG, electroencephalogram; IED, interictal epileptiform discharge; RT, reaction time.

In iART, IED‐bursts led to incorrect responses in four patients (20%) and missed responses in four patients (20%), but both conditions were only observed in two patients (Table 2, Patients 6 and 7). The per‐person probabilities for incorrect responses and missed responses differed between IED‐bursts (mean 2.3%/median 0% and mean 3.0%/median 0%, respectively) and normal EEG (mean .5%/median 0% and mean 0%/median 0%) but did not reach statistical significance (*p* = .13 and *p* = .13, Wilcoxon test; Table 2, columns 11, 10). IED‐bursts were not associated with significantly more incorrect responses (mean 1.8%, median 0) than missed responses (mean 3.0%, median 0; *p* = .56, Wilcoxon test) after correction for incorrect and missed responses during normal EEG (Table 2, columns 14, 15, and Table S2, columns BC, BD).

**TABLE 2:** Test results of the prospective pilot study: Using interictal Automated Responsiveness Test.

1	2	3	4	5	6	7	8	9	10	11	12	13	14	15	16	17	18
Patient	Test duration, min	Triggers, *n*	IEDs, *n*	Incorrect response during normal EEG, *n*	Missed response during normal EEG, *n*	Correct response during IED, *n*	Incorrect response during IED, *n*	Missed response during IED, *n*	Incorrect response during normal EEG, %	Missed response during normal EEG, %	Incorrect response during IED, %	Missed response during IED, %	Incorrect response due to IED‐bursts, %	Missed response due to IED‐bursts, %	IED‐ detection sensitivity	IED‐ detection specificity	False‐positive IED detection rate/min
1	N/A for technical reasons																
2	10	66	51	0	0	50	1	0	.0	.0	1.9	.0	1.9	.0	.94	.99	1.5
3	N/A for technical reasons																
4	N/A for technical reasons																
5	9	25	4	0	0	4	0	0	.0	.0	.0	.0	.0	.0	.80	.99	2.3
6	21	91	10	2	1	7	2	4	2.6	.0	12.5	25.0	9.9	25.0	.88	.98	4.1
7	16	45	12	0	0	10	1	1	.0	.0	8.3	8.3	8.3	8.3	.82	.99	2.1
8	29	73	1	1	0	1	0	0	1.4	.0	.0	.0	−1.4	.0	.50	.99	2.5
9	26	33	21	0	0	21	0	0	0	0	0	0	0	.0	.63	.99	.5
10	52	61	0	0	0	0	0	0	0	.0	0	.0	.0	.0		.99	1.2
11	23	140	60	2	0	59	0	1	2.8	0	0	1.7	−2.8	1.7	.96	.99	3.5
12	N/A because of cognitive impairment of patient																
13	20	105	67	0	0	67	0	0	0	0	0	0	0	.0	.98	.99	1.9
14	29	79	29	0	0	29	0	1	0	0	0	3.4	0	3.4	.93	.99	1.6
15	31	33	12	0	0	12	0	0	0	0	0	0	0	0	.91	.98	1.7
16	N/A, no indication for test from referring physician																
17	N/A, no indication for test from referring physician																
18	N/A, no indication for test from referring physician																
19	26	66	5	0	0	5	0	0	0	0	0	0	0	.0	.83	.99	2.3
20	13	60	28	0	0	26	2	0	0	0	7.1	0	7.1	.0	1	.99	3.2
Mean	23.5	67.5	23.1	.4	.1	22.4	.5	.5	.5	.0	2.3	3.0	1.8	3.0	.85	.99	2.2
SD	10.8	30.6	21.9	.8	.3	21.9	.7	1.1	1.0	.0	4.0	6.8	3.8	6.8	.14	.00	.9
Count	13	13	13	12	12	13	13	13	13	13	13	13	13	13	12	13	13
Median	23.0	66.0	12.0	.0	.0	12.0	.0	.0	.0	.0	.0	.0	.0	.0	.90	.99	2.1
Median (95% CI)									0 (0–1)	0 (0–0)	0 (0–7)	0 (0–3)	0 (0–7)	0 (0–3)	.90 (.80–.96)	.99 (.99–.99)	2.1 (1.5–3.2)

*Note*: Column 1: The tested patients whose medical history is described in Table S2. Column 2: The duration of a session with iART in minutes. Column 3: The total number of triggers generated by the electronic circuit of iART based on the IED‐burst detection by the model and identified as true‐positive and false‐positive triggers in the offline analysis. Column 4: The number of IED‐bursts detected by the model that were visually confirmed as true positive triggers in the offline analysis. Column 5: The number of incorrect responses during normal EEG in a session. Column 6: The number of missed responses during normal EEG in a session. Column 7: The number of correct responses in a session that were associated with IED‐bursts. Column 8: The number of IED‐bursts in a session that were associated with an incorrect response. Column 9: The number of IED‐bursts in a session that were associated with a missed response. Column 10: The probability of incorrect responses during normal EEG is the number of incorrect responses during normal EEG in relation to all triggers by the model during normal EEG in one session, given in percent. Column 11: The probability of missed responses during normal EEG is the number of missed responses during normal EEG in relation to all triggers by the model during normal EEG in one session, given in percent. Column 12: The probability of incorrect responses during IED‐bursts is the number of measured incorrect responses associated with IED‐bursts relative to the number of all IED‐bursts in one session, given in percent. Column 13: The probability of missed responses during IED‐bursts is the number of measured missed responses associated with IED‐bursts relative to the number of all IED‐bursts in one session. Column 14: The probability of incorrect responses due to IED‐bursts is the IED‐associated incorrect response probability (column 12) adjusted for the percentage of incorrect responses during normal EEG (column 10) in one session (i.e., subtracted from each other). Column 15: The probability of missed responses due to IED‐bursts is the IED‐associated missed response probability (column 13) adjusted for the percentage of missed responses during normal EEG (column 11) in one session (i.e., subtracted from each other). Column 16: Sensitivity of the model to detect IED‐bursts in the EEGs recorded together with iART. Column 17: Specificity of the model to detect IED‐bursts in the EEGs recorded together with iART. Column 18: False‐positive IED‐detection rate in iART, calculated as the number of false‐positive triggers (by the model during normal EEG) divided by the duration of the test session in minutes.

Abbreviations: CI, confidence interval; EEG, electroencephalogram; IED, interictal epileptiform discharge; N/A, not available.

The well‐known association between electrophysiological IED‐burst characteristics and their clinical correlates was confirmed (Appendix S1, p. 22).^e.g., 2^ In the clinical pilot study, in which only one EEG‐channel was used for IED‐burst detection for technical reasons, it was shown that the anatomical‐functional specificity of IED‐bursts was quite well preserved, that is, the actual prevalence of incorrect and missed responses in iART was measured for most patients (Appendix S1, p. 23). Research reports (Appendix S1, p. 31) were prepared for each patient, sent to the referring physician, and shared upon request with the patients, providing an option for patients' empowerment.

Table S2 (columns AI‐AM and BI‐BM) shows the percentages of true‐positive, true‐negative, false‐positive, and false‐negative detections by the model in relation to the total number of (MTF‐image) classifications per test session. The median sensitivity and specificity of IED‐burst detection did not differ between the car test (Table 1, columns 15, 16) and iART (Table 2, Columns 16, 17; *p* = .45 and *p* = .11, respectively, Wilcoxon test). The median false‐positive IED‐detection rates were 3/min (95% CI = 2–6; Table 1, column 17) in the car test and 2/min (95% CI = 2–3; Table 2, column 18) in iART (*p* = .02, Wilcoxon test).

## 4 DISCUSSION

Our artificial intelligence‐based system detected IED‐bursts with variable appearance in real time and comprehensively assessed their impact. This has not been possible until now due to the lack of versatile, objective, rapid, and automatic methods. In this study, we demonstrated the functionality of DigRTEpi in quantifying the degree of impaired brain function related to IED‐bursts by assessing virtual driving performance, which could potentially contribute to road safety. As our youngest patient was 11 years old, DigRTEpi can also be used to evaluate traffic behavior like biking for children. DigRTEpi can also determine whether IED‐bursts are impairing cognition, memory, and social interactions. The results of iART in our clinical pilot study support this statement. The information obtained by DigRTEpi will help guide diagnosis and treatment decisions of referring physicians and empower PWE to better understand their situation.

### 4.1 IED‐detection model and its optimizations

The model is based on the idea that different EEG patterns have different relationships between adjacent microvolt values. The relationships are taken from the frequency relationships between the amplitude quantiles (i.e., the number of values in the quantiles), into which the raw EEG is initially mapped and described as transition probabilities. MTFs visualize transition probabilities between individual microvolt values using different patterns of pixels of varying intensity. MTFs in combination with a deep neural network have been successfully applied to classify nonmedical data.^18^, ^19^ Medical data like electrocardiograms and electromyograms were successfully classified using a Markov transition matrix or MTFs,^20^, ^21^ but MTFs have not been applied yet to classify IEDs in EEGs.

We improved model performance by replacing the global cross entropy loss function of ResNet34 with a focal balanced CE loss function.^16^ Although we trained the model with a 5:1 ratio of normal EEG to IED‐bursts, the imbalance between normal EEG and IED‐bursts ranged from 7:1 (Figure 4B) to ≈30:1 (Figure S2B) in our data, and classification performance was still high. It was shown that unsupervised pretraining of a neural network improved deep learning.^22^ We used two supervised learning strategies (using hand‐labeled data) for pretraining, and model performance still improved. The mechanisms behind classification improvement may be similar: pretraining establishes a lower local minimum in the loss function from where the model can apparently better generalize. The generalization error, for example, a drop in classification performance of new EEGs, was low. Sensitivity and specificity in the prospective pilot study tended to be higher than with the test segments of the EEG dataset that was used to train the model (90% and 99% compared to 84% and 96%).

### 4.2 Comparison with automated EEG‐analysis systems

Compared to other systems, including time–frequency methods,^e.g., 23^ the half‐wave approach using geometric methods,^24^ statistical signal processing methods,^25^ and deep learning using all (raw) microvolt values for feature extraction and classification,^26^, ^27^, ^28^, ^29^, ^30^, ^31^, ^32^, ^33^, ^34^, ^35^, ^36^, ^37^, ^38^, ^39^, ^40^, ^41^, ^42^, ^43^, ^44^ our IED‐detection model together with the closed loop has several advantageous features. The short classification latency, which may be due to the MTF technology with relatively small computational effort, enables real‐time IED‐burst detection, allows IED‐bursts to trigger tasks, corrects for the closed loop's digital latency, and permits transient IED‐effect measurements. IED‐bursts of variable morphology are detected with high sensitivity and specificity in focal and generalized epilepsies, enabling the use of DigRTEpi in clinical routine. We have previously shown that focal IED‐bursts with a duration of <1000 ms, which also included single IEDs, had an effect.^2^ The minimum duration of focal IED‐bursts detected by DigRTEpi is 400 ms. These are likely to be single IEDs. However, single IEDs were in the minority in this dataset. DigRTEpi combines the assessment of behavior (car test) and cognitive function (iART) in one system. All these features are important, because there is still disagreement about a suitable/ideal test to measure IED‐effects.^6^

### 4.3 Possible future of IED criteria

DigRTEpi detected clinical correlates of IED‐bursts in the car test and iART that ranged from mildly slowed or incorrect responses to more severe, missed responses. Effect variability of IEDs has been observed before. Therefore, the traditional distinction between IEDs and seizures has been repeatedly questioned.^45^, ^46^ This boundary is exemplified by our Patient 6 (Table S2), who had absence epilepsy and impaired responses during generalized spike–wave discharges (mean duration = 5234 ms, min = 1297, maximum = 9171). Are these electroclinical events seizures or IED‐bursts? It was suggested earlier that “the slowing of a response time or an error made during testing should be considered evidence of a seizure when accompanied by appropriate E.E.G. changes, even though nothing has been detected by routine clinical observation.”^45^ The IED criteria could be simplified using DigRTEpi by adding a functional component that divides IED‐effects into minor or clinically relevant and frees the IED criteria from meeting strict morphological EEG‐definitions. A starting point could be to define the laboratory measurements of missed responses as clinically relevant, or if not measured, to predict their risk from the extent of RT prolongation, as we have previously suggested.^2^ There is probably a continuum between interictal and ictal rather than a sharp boundary, perhaps analogous to the critical care setting, where there is extensive recent literature and formal definitions of the ictal–interictal continuum.^13^, ^47^ By objectively and systematically assigning clinical correlates to EEG abnormalities, DigRTEpi can improve IED criteria and help personalize care.

### 4.4 Clinical considerations

Our original intention was to make DigRTEpi freely available. To ensure that PWE have broad access to DigRTEpi, the next steps regarding technical and clinical applicability should be taken by a few people. We have therefore patented DigRTEpi. We plan a user‐friendly, affordable, successor model with fully automated analysis and reporting that can also be used in outpatient clinics with limited technical expertise and financial resources.

DigRTEpi offers objective and standardized assessment of IED‐burst effects as minor or clinically relevant. Should the measurement of relevant effects have therapeutic consequences? The following situations could be examples of the best way to treat the patient by “treating the EEG”: Left temporal IEDs or IED‐bursts associated with speech arrest, frequent IED‐bursts in children and adolescents with epilepsy, as IEDs without seizures were shown to lead to long‐lasting synaptic disorganization and impaired neurogenesis in the developing human brain and in animal models,^48^, ^49^ or IED‐bursts with electrophysiological characteristics in certain occupations, such as professional drivers,^2^ where IEDs are considered (in Switzerland, IEDs are considered for all road users). Longitudinal studies are needed to see whether treatment of IEDs or IED‐bursts helps, and DigRTEpi also offers the possibility of monitoring changes in IED‐burst effects. These measurements should be accompanied by a questionnaire on quality of life in epilepsy.^50^ The goal of established neuropsychological tests is to assess the general level of cognitive function in people with epilepsy, for example, during presurgical evaluation. These tests are not timed to coincide with the appearance of IED‐bursts, or that IED‐bursts themselves trigger neuropsychological tasks. In addition, many tasks are complex and require some time to be completed. IED‐bursts of short duration and particular location may increase not the error rate but only the time required to complete the task. Thus, many established neuropsychological tests are not well suited to detect IED‐induced deficits. iART integrated into DigRTEpi was specifically designed to detect IED‐associated TCI.

### 4.5 Limitations of DigRTEpi

Limitations of DigRTEpi include high false‐positive IED‐detection rates, limitation of IED detection to one channel, manual scoring of iART, and manual statistical analysis of test results. Interrater reliability was not reported using a kappa statistic, because the focus was on determining the effects of IEDs of various types. In our follow‐up project, we aim to develop and validate EEG personalization code to reduce the false‐positive IED‐detection rate, extend IED detection to all channels of a 10–20 recording by introducing an MTF‐image pooling layer before the ResNet backbone, accelerate the detection of IEDs with larger amplitude spike/sharp components by reducing the sampling bandwidth to a Nyquist frequency as low as 64 Hz, replace manual iART scoring with automated procedures, and implement a decision tree algorithm that will determine whether IED‐associated measures of cognitive impairment and reaction slowing are clinically relevant to facilitate the differentiation between IED‐bursts and seizures.

## AUTHOR CONTRIBUTIONS

Andreas von Allmen designed and conceptualized study, built custom‐made devices for the study, and revised the manuscript for intellectual content. Diyuan Lu analyzed the data, interpreted the results, and revised the manuscript for intellectual content. Caroline Jagella analyzed the data, interpreted the results, and revised the manuscript for intellectual content. Yasmina Abukhadra analyzed the data, interpreted the results, and revised the manuscript for intellectual content. Rune Markhus assisted with patient recruitment and phenotyping, interpreted the results, and revised the manuscript for intellectual content. Jochen Triesch interpreted the results and revised the manuscript for intellectual content. Margaret Gopaul interpreted the results and revised the manuscript for intellectual content. Lawrence J. Hirsch conceptualized the study, analyzed and interpreted the results, and revised the manuscript for intellectual content. Felix Rosenow assisted with patient recruitment and phenotyping, interpreted the results, and revised the manuscript for intellectual content. Hal Blumenfeld designed and conceptualized the study, analyzed and interpreted the results, and revised the manuscript for intellectual content. Heinz Krestel designed and conceptualized the study, analyzed the data, drafted the manuscript for intellectual content, acquired funding, had full access to all the data in the study, and takes responsibility for the integrity of the data and the accuracy of the data analysis.

## FUNDING INFORMATION

This study was supported by the European Union's Framework Program for Research and Innovation Horizon 2020 (2014–2020) under Marie Sklodowska‐Curie grant agreement No. 99791.

## CONFLICT OF INTEREST STATEMENT

H.K. was supported by the European Union's Framework Program for Research and Innovation Horizon 2020 (2014–2020) under Marie Sklodowska‐Curie grant agreement No. 99791. A.v.A. and H.K. filed DigRTEpi for patent. None of the other authors has any conflict of interest to disclose. We confirm that we have read the Journal's position on issues involved in ethical publication and affirm that this report is consistent with those guidelines.

## Supporting information


Figure S1.

Figure S2.

Table S1.

Appendix S1.

Video S1.

## Data Availability

The data that support the findings of this study are available from the corresponding author upon reasonable request.
