# Supplementary figures and images for: Digital Response Test in Epilepsy assesses interictal epileptiform discharge effects in real time
Source: Epilepsia. 2025 Oct 3;67(1):381–95. doi: 10.1111/epi.18629 (PMC12893249; doi:10.1111/epi.18629)

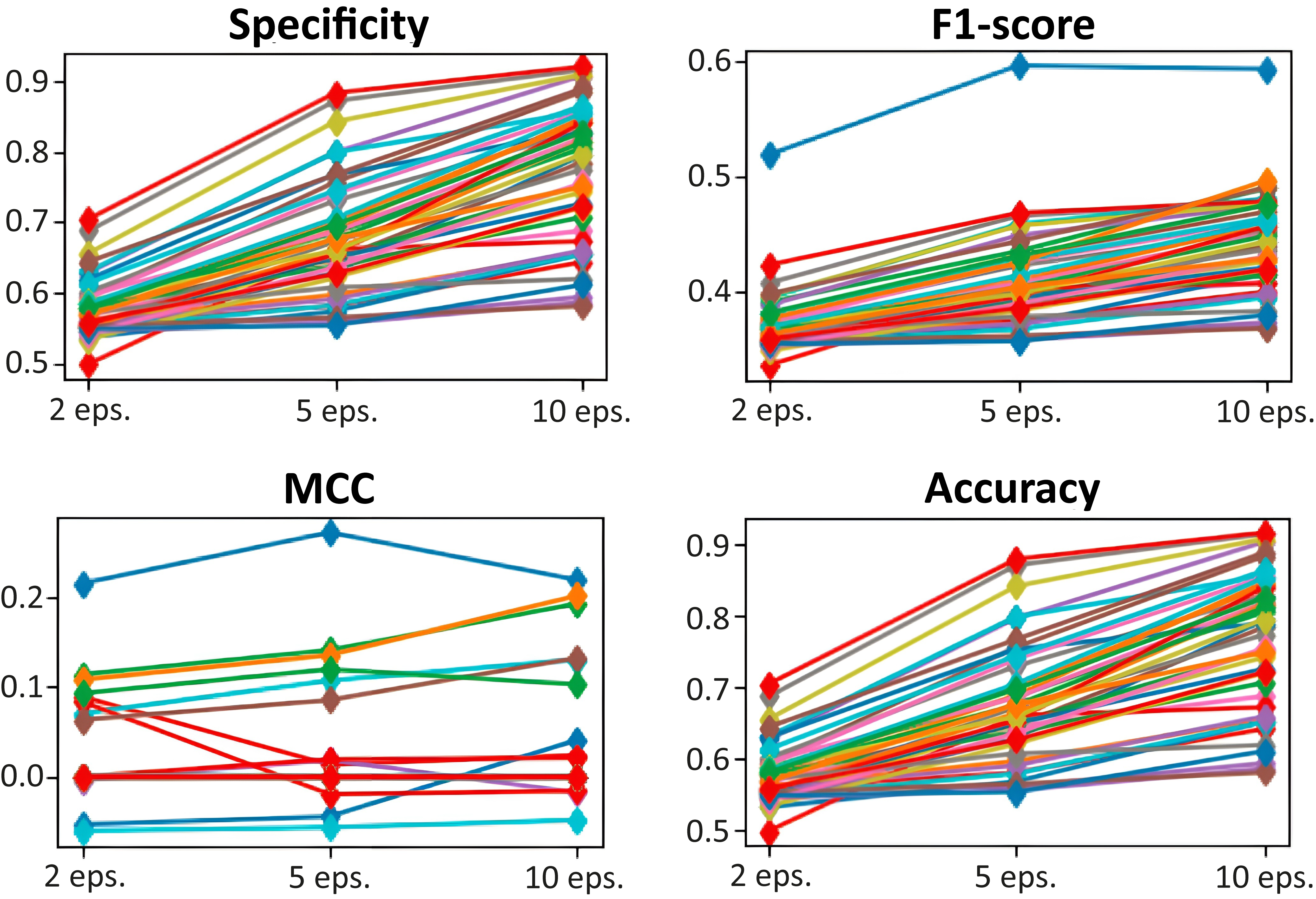

Supplement: Supplementary file 1 — Figure S1. [file EPI-67-381-s003.tif]

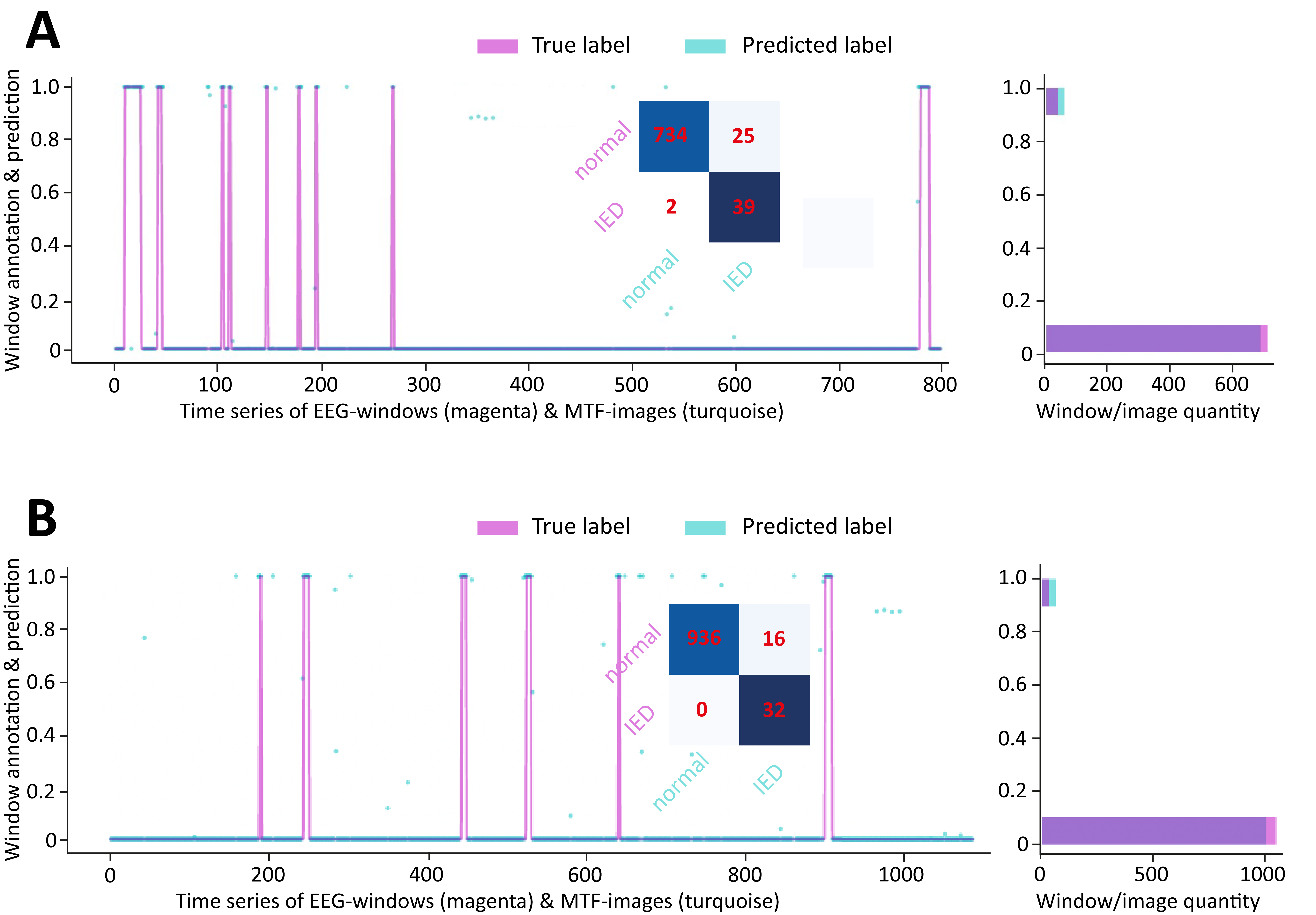

Supplement: Supplementary file 2 — Figure S2. [file EPI-67-381-s002.tif]

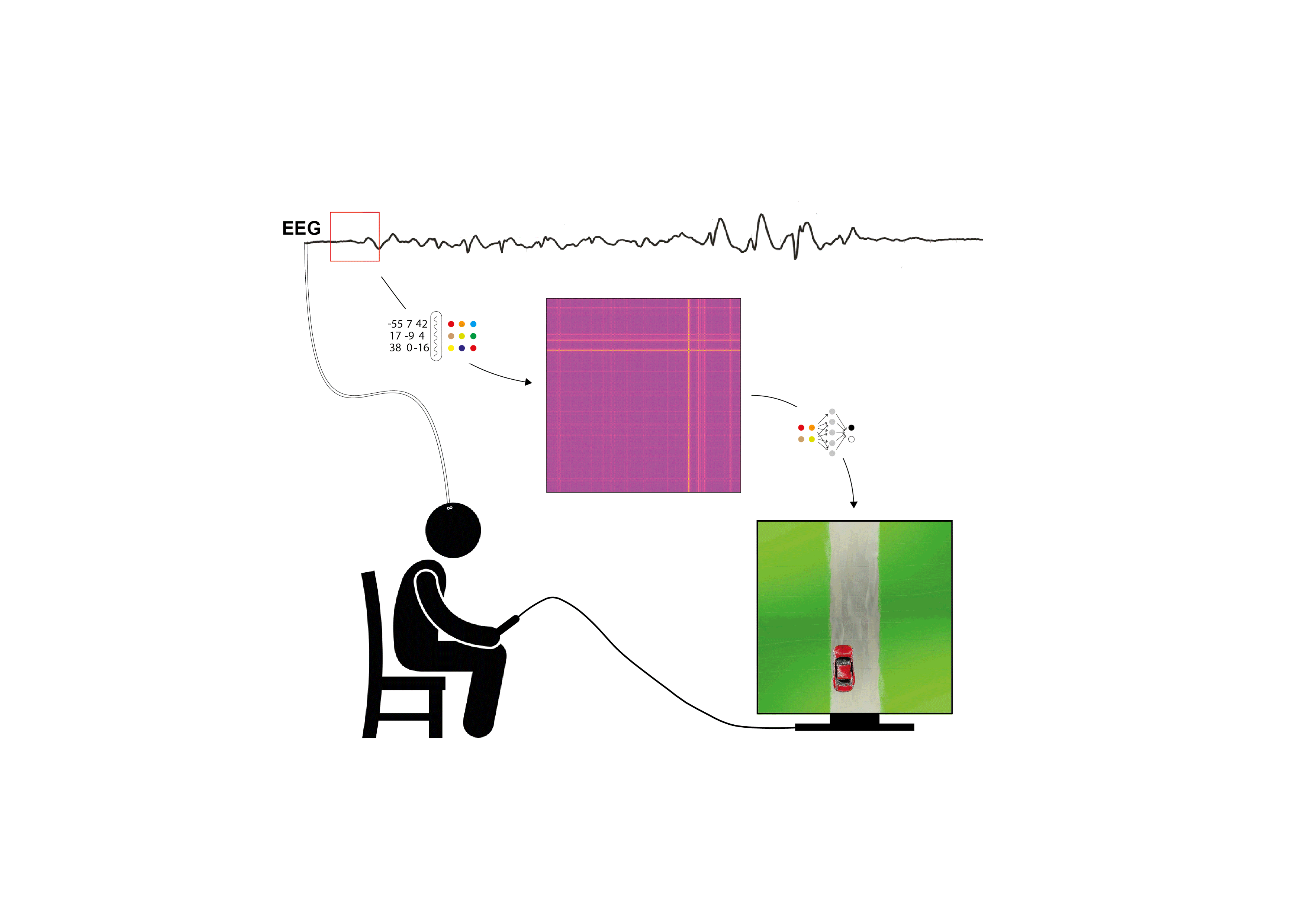

Supplement: Supplementary file 5 — Video S1. [file EPI-67-381-s001.gif]
