# Supplementary material for: Digital Response Test in Epilepsy assesses interictal epileptiform discharge effects in real time
Source: Epilepsia. 2025 Oct 3;67(1):381–95. doi: 10.1111/epi.18629 (PMC12893249; doi:10.1111/epi.18629)
Supplement: Supplementary file 4 — Appendix S1. [file EPI-67-381-s004.docx]

**Appendix S1**

Methods: p. 1

Results: p. 19

Discussion: p. 33

**Methods**

**The EEGs used for training, optimization, and testing the IED-detection model**

All the EEGs from Bern University Hospital and the National Epilepsy Centre Oslo were recorded together with reaction tests. Since the authors RM, AvA, and HK had already worked together prior to this study, many of the reaction tests are similar. The flash test used during EEG-recordings from Bern and Oslo were identical in that patients with their eyes closed responded to single flashes from a flickering light. The car tests used during the EEG-recordings from Bern and Oslo were comparable but not identical. The car test in Oslo had the added feature of divided attention. But otherwise, author RM obtained the car test as seen in Figure 1B and Video S1 from the authors AvA and HK. The cognitive test used during the EEG-recordings from Oslo was self-developed and named Multi-Aspect Perceptual Awareness test (MAPA). MAPA measured reaction times for stimuli presented above, below, left, or right in relation to a fixation cross. It also measured the accuracy of responses and the patients’ awareness of the accuracy of their own responses. The commercial PC-based realistic driving simulator software used during the EEG recordings from Oslo was from Carnesoft, The Netherlands.

The scalp EEGs used for supervised learning were recorded in the proprietary format of the EEG acquisition systems that were used by the three epilepsy centers. In Bern and Oslo, EEG-acquisition systems from Nicolet (acquired by Natus) were used, and in Frankfurt, EEG-acquisition systems from Micromed (acquired by Natus) were used. When exported in EDF format, each EEG was de-identified and given an alpha-numeric code. When loaded into EEG viewer software, each EEG was filtered with a bandpass between 1 and 30 Hz and a 50-Hz notch filter to reduce artifacts due to motion, eyes, and muscle. In each EEG displayed in longitudinal bipolar montage, one channel was identified that most clearly showed the IED-bursts and had the fewest artifacts. Two experienced epileptologists (HK, CJ) and one computer science student (YA, under supervision) used this channel to manually label the beginning and end of each individual IED-bust and record their time. IED-burst duration was automatically calculated.

**Digital Response Test in Epilepsy (DigRTEpi)**

DigRTEpi was patented.^51^

Communication protocols

We received software development kits (SDKs) from the EEG companies Natus and Micromed (before Natus acquired Micromed) that we configured for DigRTEpi. Both Micromed and Natus use a TCP/IP protocol for a packet-switching network, although there are differences. Micromed's EEG-acquisition PC serves as a TCP/IP client and provides EEG-packets, while the study laptop acts as a server and receives EEG-packets. We implemented the client part of the protocol for the study laptop in the Python programming language and placed the server part of the protocol on an USB stick so that EEG data could be transmitted from different EEG-acquisition PCs. Ethernet LAN switches were installed to communicate between the study laptop and the EEG-acquisition PCs from Micromed at the Epilepsy Center Frankfurt Rhine-Main, where one arm of the prospective diagnostic case series was performed.

The solution for the EEG transmission in the second arm of the prospective pilot study at the Yale Comprehensive Epilepsy Center, using a Natus acquisition station, was different. Natus also uses a TCP/IP protocol, but the open-source high-performance message library ZeroMQ (<https://zeromq.org/>) has been implemented for packet switching. It queues messages or packets but does not use a message broker (a program module for message validation, transformation, and forwarding) to transmit them, hence the zero in ZeroMQ for "zero broker". The lack of a message broker makes this messaging library faster than traditional packet-switching networks. ZeroMQ uses a "publish-subscribe" pattern of data transfer instead of a "server-client" mechanism with the EEG acquisition station being the “publisher”. This communication protocol was used between the XLTEK EEG acquisition stations installed at Yale New Haven Hospital and the study laptop.

In principle, individual microvolt values were continuously streamed in small packets using a communication protocol between the EEG-acquisition PC and the study laptop. We used the sliding window technique to analyze the EEG portion-by-portion. The IED-detection model always processed 200 microvolt values, or one window, at once. The window moved with the EEG recording at a step size of 50 microvolt values. As soon as the study laptop had received 50 new microvolt values, they were pooled with 150 microvolt values from the previous window and immediately transformed and classified by the model. As the processing (i.e., the transformation and classification) of one window took a median 98.7 ms (see Results), the model had to wait about 96 ms $(195 ms (stepsize\approx time for streaming 50 new data points)-98.7 ms (median latency to process a window\approx200 data points)=96.3 ms)$ before processing the content of a new window. Taken together, the model waited for about 100ms to receive 200 microvolt values and then processed each window in approximately 100 ms.

Input and output of the IED-detection model

When 50 new microvolt values were received by the laptop, the model first downsampled the data to 256 Hz and filtered them with a bandpass between 1 and 30 Hz and a 50 Hz notch filter (60 Hz in the U.S.). Second, it pooled the 50 microvolt values with 150 microvolt values from the previous window. Only then did the model transform the 200 microvolt values in an image using Markov Transition Fields and classify the image using ResNet. The output of the IED-detection model was the activation of an application programming interface called pylibftdi, a library that came with the transistor-transistor-logic chip (TTL) (Future Technology Devices International) and was installed on the study laptop.

Electronic circuits of the closed-loop circuit

The first electronic circuit was a transistor-transistor-logic chip (TTL) (Future Technology Devices International) that gated the signal from pylibftdi once the model classified an MTF-image as an IED-burst. The TTL-chip either activated the car test or the interictal Automated Responsiveness Test (iART), a new cognitive assessment (see below). The printed circuit boards for the two tests had to be changed in the closed-loop circuit, depending on which test was used to assess the patients. All electronic circuits were built by AvA.

Electronic circuit of the car test

The electronic circuit had several connections. The first input on the printed circuit board came from the TTL-chip via a USB-C jack connection. A 100 ms TTL-signal activated this electronic circuit which in turn triggered an obstacle on the road of the car test. Activation of the electronic circuit also synchronized the triggering of the obstacle with the ongoing EEG-recording via a DC output of the board to the amplifier/headbox of the EEG-acquisition station using a cable with a jack connection on one end and two pins on the other end (3.5mm jack to 2-pin touch-proof DIN connector with resistor bridge). The signals sent were DC voltages (0 – 2.5V), displayed as a square wave signal in an empty channel of the EEG-recording as soon as an IED-burst was detected. For a clean signal, filtering of this channel (as was done for the EEG channels) had to be disabled. The rise of the square wave signal indicated the activation of the electronic circuit of the car test. The time from the activation of the car test’s electronic circuit to the appearance of the obstacle on the road was variable. This digital latency was measured and bypassed using a photo-voltaic sensor attached to the laptop monitor at the top edge of the driving videogame where the obstacle appeared. Obstacle appearance changed the light/dark contrast and activated the photo-voltaic sensor, which in turn started a stopwatch of the electronic circuit to measure reaction times that were free of the digital latencies of the circuit (so-called effective reaction times). The photo-voltaic sensor was connected to the board with a jack connection. The reaction time measurement was stopped when a patient pressed a push-button that was also connected to the board via a separate input using a jack connection. The printed circuit board contained a microcontroller that ran the Linux operating system.

Car test software

The Java application was written by AvA. It displayed a car on a straight road and an obstacle that came down the road when it was triggered. The car could not swerve, and the patient had one second to change lanes by pressing a button, otherwise the car crashed into the obstacle. The duration of 1 second was programmed based on attention lapses (which can also be recorded with the car test during normal EEG) and microsleep, the shortest duration of which, depending on the literature, is half a second to a second and which are known to contribute to accidents in real life. The software automatically measured the reaction times, registered the missed reactions, that is, the crashes, and saved the log file with reaction times and crashes in the root directory of the car test on laptop’s hard disk.

Electronic circuit of the interictal Automated Responsiveness Test (iART)

The car test as well as iART were developed for patients eight years and older. iART was named after the “Automated Responsiveness Testing in Epilepsy” (ARTiE) from which it was developed.^52^ iART’s printed circuit board contained a microcontroller (adafruit) that was galvanically isolated from the DC input for the signals from the TTL-chip. As on the circuit board of the car test, an input signal from the TTL-chip came via a USB-C to jack connection and activated this electronic circuit. The input signal was emulated as a USB keyboard command by the microcontroller and sent to the laptop as a TTL-trigger through a USB interface. This activated video playback on the study laptop.

The activated electronic circuit also sent a signal via a DC output to the amplifier/headbox of the EEG-acquisition station to synchronize the triggering of a video with the ongoing EEG recording. A cable with a jack connection on one end and two pins on the other end was used for this connection (see car test). When an IED-burst was detected by the model, a DC signal was displayed as a square wave in an additional channel of the EEG recording, and the rise of the square wave corresponded to the activation of the electronic circuit. The digital latency of the closed-loop circuit was not corrected for iART because the patient responses to the videos that assessed cognitive functions were not measured with millisecond resolution.

iART software

iART used the freely available PsychoPy software (<https://www.psychopy.org/>).^12^ A script in the Python programming language on the study laptop started video playback when it was activated by a TTL-trigger from the iART circuit board (i.e., after an IED-burst was detected). The Python script included a random number generator that arbitrarily picked one of the 40 videos, or the audio sound that played white noise, which had been assigned a specific number (Table S4). Only the videos that tested memory and executive function were played in a specific order. The videos contained brief instructions on video, acoustic and written information (subtitles). They lasted up to 5 seconds and tested orientation (time, person, place), language comprehension, word recall, word repetition, knowledge of body parts, left-right discrimination, apraxia, memory (e.g., time), number comprehension, and executive function. The executive function was tested by playing the video “Say” and a video randomly showing one of 24 numbers, followed by the video “Say” and white noise from the audio file. We have recorded the instructions in 17 different languages: Arabic, Chinese, Croatian, Czech, English, French, German, Greek, Italian, Lithuanian, Norwegian, Polish, Portuguese, Romanian, Russian, Spanish, and Turkish. These instructions were recorded with native speakers using Zoom Video Communications software. The video-audio files were saved in mp4 format and professionally edited (AllmenFilms, Switzerland). The test battery can be easily expanded by recording new videos with new tasks and in new languages.

**IED-detection model**

**Markov Transition Fields**

A (first order) Markov chain or Markov process is a stochastic model that describes a sequence of possible events in which the probability of each future event depends only on the current state that has been reached. Thus, no additional information from the past is required for a prediction. Markov chains have many applications as statistical models for real-world processes such as predicting stock market prices, currency rates, or in gambling. Markov processes form the basis for general stochastic simulation methods, for example in Markov chain Monte Carlo methods and reinforcement learning. Markov Transition Fields (MTFs) visualize the relationships of the data points of a time series.^20,53^ Data points are first discretized into quantiles based on their microvolt value or amplitude. By discretizing data points, the time series is also compressed, which can be advantageous for large time series and to reduce the computational effort. A Python Package for Time Series Classification using Markov Transition Fields is publicly available: <https://github.com/johannfaouzi/pyts>.^54^ We used the EEG as a continuous time series of microvolt values together with the sliding window technique to transform the EEG window-by-window and subsequently classify these portions. All microvolt values from each EEG window were assigned to a first matrix, the Markov transition matrix. Assignment was based on the absolute size or amplitude of each microvolt value into 32 evenly spaced quantiles (quantiles $q_{i}$ on the column side, and quantiles $q_{j}$ on the row side $(i, j\mathbb{\in N,}\left[ 1, 32 \right])$). The size of the quantiles was recalculated for each window to account for EEG amplitude fluctuations from one window to the next. The transition frequencies between the quantiles were obtained by dividing the number of microvolt values in each of the 32 quantiles by the number of microvolt values in each row of the matrix. The transition frequencies or weights $w_{ij}$ were normalized using the equations $w_{0}^{1}=\frac{w_{ij}-w_{min}}{w_{max}-w_{min}}$ and $\sum_{j} w_{ij}=1$. The transition probabilities (normalized $w_{ij}$) from the Markov transition matrix were assigned to each consecutive pair of the 200 microvolt values from each window, resulting in a Markov Transition Field matrix with size 200 x 200. The transition probability for each pair of the 200 microvolt values was plotted in an image that we called Markov-Transition-Field image or MTF-image (size 200 x 200 pixels). The brightness of a pixel indicated the (inverse) probability of a transition from quantile (y1,x1) to quantile (y2,x2) (1 and 2 are points in a time series at the time x1 and x2). in other words, brighter pixels indicate higher probabilities for pairs of microvolt values to have different amplitudes (i.e., to transition between amplitude quantiles). An MTF-image was symmetrical along the diagonal, which represented the self-transition probability.

**Residual Convolutional Neural Network (ResNet)**

Each MTF-image was not saved by default but immediately classified by ResNet34,^55^ a freely available residual convolutional neural network (CNN) with 34 neural layers and skip connections that provide deep neural networks with the flexibility to learn representations more efficiently, especially in image classification tasks. We replaced the default ResNet34 output layer with 1000 output units and softmax activation by a two-unit dense layer with sigmoid activation function for binary classification, i.e., for classification of MTF-images representing IED-bursts or normal EEG. We used randomly initialized weights to train ResNet34 with the EEGs from the three epilepsy centers. To compare the classification performance, we downloaded the freely available ResNet34,^17^ which had been pre-trained in a supervised manner with ImageNet.^56^ Pre-trained ResNet was subsequently retrained with the EEGs from the three epilepsy centers (see Appendix S1, Optimization of IED-detection model, p. 14).

**Data preparation for the IED-detection model**

For each EEG used to train the model, the channel identified for IED-burst labeling was segmented and its individual microvolt values or data points were labeled “1” if within the IED-beginning and -end times and “0” if outside the IED-burst marks. The data points and their labels [0,1] were divided in training, validation, and test segments based on a ratio of 70:15:15. The labels [0,1] were summed for each window. If the sum was ≥ 150 (equal to or more than ¾ of the maximal score), the window and subsequent MTF-image was defined as an IED-burst. Conversely, a window with a sum of < 150 was defined as (as a segment of) normal EEG. A sum of ≥ 150 per window corresponded to ≥ ¾ of the transition probabilities $w_{ij}$in the MTF-matrix as observed in IED-bursts. These ≥ ¾ of the IED-typical transition probabilities appear in the MTF-images as characteristic patterns and pixel intensities. In this way, hand-labeled IED-burst annotations were converted into MTF-image information that the model could learn to distinguish between IED-bursts and normal EEG. The training segments of all EEGs (from Bern, Frankfurt, and Oslo) were combined to train the model for 100 epochs. The Adam optimizer with a learning rate of 0.0005 (without learning rate decay) and a mini-batch size of 64 was used for gradient back-propagation. The validation segments of all EEGs from Bern, Frankfurt, and Oslo were used for optimizing model performance. The test segments were used to determine the classification performance of the model after we considered it fully optimized. Classification performance of the fully optimized model was determined based on i) all test segments from Bern, Frankfurt, and Oslo together (concatenated); ii) for all test segments of Bern, Frankfurt, and Oslo separately (site-specific classification performance; data not shown); and iii) the test segments of individual EEGs from Bern, Frankfurt, or Oslo (Figure 4A, Figure S2A and S2B).

**Coding environment of the IED-detection model**

Our model was built in a conda environment with tensorflow 2.2.0, keras 2.3.0, and python 3.8.5 and was trained on a Windows machine with an Intel^®^ Core160 i7-4770 CPU, 16 GB RAM and a GeForce GTX2080 GPU with 8GB of memory.

**Optimization of the IED-detection model**

Experiments with MTFs

We experimented with the size of the (first) Markov Transition Matrix by reducing or increasing the number of quantiles into which the EEG was discretized. The default size of 32x32 quantiles was ultimately retained because changing the matrix size did not improve model classification performance (data not shown). We also experimented with the size of the window, that is, the number of microvolt values that the MTFs transformed and ResNet classified at once. Using between 200 and 800 microvolt values, the window size was set to 200 data points at 256 Hz sampling frequency. The corresponding duration of one window was 0.781 s ($\frac{200 (data points)}{256\frac{1}{s}(sampling frequency)}=0.781 s)$. The window moved forward with the ongoing EEG-recording at a step size of 50 data points. 50 datapoints corresponded to $\frac{50}{256 \frac{1}{s}} =0.195 s$ at a sampling frequency of 256 Hz. Thus, the step size was about 200 ms and each window overlapped with the next window by about 150 datapoints or 600 ms. The reasons for this window size were, *first*, the minimum duration of our target structure, the IED-burst (in <<5% it was a single IED, i.e., a spike-wave), which we had set at 0.4 s. Epileptiform EEG changes were only marked by hand if they had this minimum length. This duration corresponded to $0.4 s \times256\frac{1}{s}=102$ microvolt values, half the number of data points in the window we defined. *Second*, to ensure that the EEG data were transmitted and processed ultra-fast. This included experimenting with the data transfer rate of the EEG communication protocol and considering the laptop's GPU, which calculated the mathematical operations of the model.

The default MTF library (python package) color-coded the pixels, but we used grayscale image arrays to reduce computational effort.

Experiments with ResNet

As single interictal spikes and IED-bursts are usually much rarer than segments with normal EEG in people with epilepsy (PWE), there are usually far fewer EEG windows and corresponding MTF-images of IED-bursts than of normal EEG. If not corrected, this class imbalance would bias the learning ability of every model, because learning and classification positively correlates with a larger sample size. Several countermeasures have been developed to address class imbalance in general. The Matthews correlation coefficient (MCC) and the area under the precision-recall curve (PR-AUC) compensate for class imbalance during classification, that is, after an algorithm has been trained and optimized. We have included these metrics in our analysis of the model’s classification performance. **Class imbalance** can also be addressed during optimization of a machine learning model. We addressed class imbalance during optimization of the model using *three strategies:* balanced cross entropy, use of a focal loss function, and using pre-defined sample sizes. *First,* we introduced balanced cross entropy by multiplying a weighing factor for each of the two classes with the global cross entropy loss function for binary classification.^16^ The global cross entropy loss function (CE) for binary classification is:

$$CE\left( p,y \right)=-log(p); if y=1$$

$$CE\left( p,y \right)=-log(1-p); if y is otherwise$$

$$p=true probability or the true label; y=predicted value of the current model$$

This can also be written as:

$$CE\left( p,y \right)=-log(p)- log(1-p)=CE(p)=-log(p)- log(1-p)$$

Weighting factors $\alpha\in[0,1]$ for class 1 (windows transformed into MTF-images of IED-bursts) and $1-\alpha$ for class 2 (windows transformed into MTF-images of normal EEG) were applied by multiplying them with the global cross entropy loss function:

$$CE(p)=-\alpha log(p)- (1-\alpha)log(1-p)$$

In this way, the loss of the majority class was down-weighted and that of the minority class was up-weighted. In practice, $\alpha$ can be set to the inverse number of samples in class 1 or treated as a hyperparameter. While $\alpha$ balanced the importance between the windows transformed into MTF-images of IED-bursts and normal EEG, it did not distinguish between easy and difficult to classify MTF-images. *Second*, by replacing the global CE loss function with the focal CE loss function,^16^ the MTF-images that were easy to classify were weighted down and the training focused on the MTF-images that were difficult to classify. To do this, a modulating factor ${(1-p_{t})}^{\gamma}$, with $p_{t}=p if y=1; p_{t}=1-p if y is otherwise$, was multiplied with the global, balanced CE loss function, turning it into a focal, balanced CE loss function (FL):

$$FL(p)=-{(1-p)}^{\gamma}\alpha log(p)- {(1-(1-p))}^{\gamma}(1-\alpha)log(1-p)$$

or

$$FL(p_{t})=-{(1-p_{t})}^{\gamma}\alpha_{t}log(p_{t})$$

$\gamma$ is a variable focusing factor. If $\gamma=0$, this is the global, balanced CE. $\gamma$ can smoothly adjust the rate at which the easy-to-classify images are down-weighted. $\gamma=2$ worked best for the experiments. *Third*, we have experimented with predefined ratios of EEG data points (i.e., windows or MTF-images) of normal EEG and IED-bursts of 1:1 and 5:1, which were presented to the model during training, to see if we could improve the classification performance.

Optimization experiments included **transfer learning,** that is, taking advantage of a pre-trained deep neural network. The principle behind transfer learning is domain adaptation. Domains consist of a feature space and a probability distribution of these features. We hypothesized that training a deep neural network on naturalistic images (with different features than those of the EEG-windows transformed into MTF-images) could be beneficial, for example, by later overfitting the model when retrained on the EEGs from our dataset. Longer training should be reflected in improved classification performance. We took advantage of the freely available ResNet34,^17^ which was pre-trained on naturalistic images from ImageNet, a dataset of 14 million manually labeled images.^56^ These deep neural networks were then retrained with the EEGs from our dataset in the same way as the original ResNet34, with the only difference being that the original ResNet34 was trained with randomly initialized weights on MTF-images (i.e., from scratch).

**Fine-tuning** of the model in Keras was a different way of taking advantage of a pre-trained deep neural network (<https://pyimagesearch.com/2019/06/03/fine-tuning-with-keras-and-deep-learning/>). In exploratory experiments, ResNet34, which was pre-trained with the EEGs from our dataset and retrained with a subset of these EEGs, was compared with ResNet34 that was trained with randomly initiated weights (from scratch) on a subset of the EEG dataset. When ResNet34, pre-trained on all EEGs from the dataset, was fine-tuned with the first 15% of microvolt values from each of the 44 EEGs of the Bern dataset recorded during the flash test (Table S1) for 2, 5, or 10 epochs (Figure S1), the model overfit at a plateau of 0.85 (data not shown). Training ResNet34 with randomly initiated weights only with the first 15% of microvolt values from each of the 44 EEGs of the Bern dataset was worse because the model plateaued at around 0.8 (data not shown). Fine-tuning all layers of ResNet34 was superior to only retraining the last two dense layers of the network (data not shown). These exploratory experiments encouraged us to fine-tune ResNet34, which was pre-trained with naturalistic images from ImageNet and retrained with all EEGs from the dataset and combine domain adaptation (different feature space of naturalistic images vs. MTF-images) with fine-tuning (identical feature space of all EEGs from our dataset vs. an EEG subset).

We finally experimented with the network **depth** by comparing classification performance between ResNet34 and ResNet50 that were both pre-trained with naturalistic images from ImageNet. Pre-trained ResNet50 can also be downloaded for free.^17^ We also changed the **depth and type** of the neural network used in the IED-detection model from a residual convolutional neural network (CNN) with 34 neural layers and skip connections between layers to reduce overfitting to a simple CNN with 10 layers. The 10-layer CNN was then trained in the same way as the original ResNet34 with randomly initialized weights on MTF-images (i.e., from scratch). The 10-layer CNN was then tested on held out subsets of MTF-images in the same way as the original ResNet34.

**Prospective pilot study, study parameters, and statistical analysis**

Identification of eligible participants

One arm of this non-significant risk study was conducted at the Epilepsy Center Frankfurt Rhine-Main. All patients recruited to this study arm were hospitalized for pre-surgical evaluation with scalp EEG-recordings. This epilepsy monitoring unit had 8 beds, and each week new patients were monitored. The study's inclusion criterion was that the people had epilepsy with IED-bursts in previous EEGs, regardless of their medication taken. These patients were asked in person during their hospitalization whether they wanted to participate in the study. They were recruited at random. Since this was a pre-surgical evaluation, the anti-seizure medication was already reduced on the second day of hospitalization and the test using DigRTEpi took place at the 3^rd^ or 4^th^ day of hospitalization.

The second arm of the prospective study was conducted at the Yale Comprehensive Epilepsy Center. The histories and EEGs of PWEs who were referred each week for medical reasons to an outpatient EEG recording at the Fitkin Neurophysiology Laboratory at the Yale New Haven Hospital were screened 1-2 weeks in advance by author HK using the databases EPIC and NeuroWorks. When an eligible participant was identified, the referring physician was asked for permission to contact the participant. If agreed, the author HK contacted the eligible participants by phone, briefly provided study information, and asked, if the eligible participants or their legal representatives were interested to participate. When they were interested, it was agreed to provide detailed information and obtain consent/assent, when they arrive for their outpatient appointment. The youngest patient recruited was 11 years old at the time of testing (Table 1 and Table S2, No.18). The patients in the second study arm were also recruited at random.

Study parameters of the car test

The log file from each test session included the reaction times (RTs, in ms), crashes (natural numbers), timestamps when the triggers occurred (hh:mm:ss:msms), and the digital latencies (ms) of the closed-loop circuit for each trigger. These parameters were imported into Excel. The RTs and crashes were sorted into two columns according to a true-positive trigger (during an IED-burst) or a false-positive trigger (during normal EEG), which was visually verified during offline analysis. For true-positive triggers, the IED-burst duration (ms) and the trigger time (ms) from the beginning of the IED-burst to the rise of the square wave in the trigger channel were measured in the EEG review software. For each test session, the mean, standard deviation, and median of the RTs, trigger times, and IED-burst durations were calculated. The number of crashes was counted. The RT-prolongation was the mean RT during IED-bursts (true-positive triggers) minus the mean RT during normal EEG (false-positive triggers) on a session level. The IED-associated crash probability was the number of IED-associated crashes divided by the number of all IED-bursts in a test session, multiplied by 100 (%). The crash probability during normal EEG, which was attributed to inattention, was the number of crashes during normal EEG divided by the number of triggers during normal EEG on a session level. Both crash probabilities were subtracted to obtain the crash probability due to IED-bursts. A patient’s cumulative risk for a crash (virtual accident) was calculated using his or her session mean RT-prolongation due to IED-bursts and a non-linear cumulative distribution function that we have previously described.^2,57^ The IED-burst prevalence was the number of visually verified true-positive triggers divided by the test duration in minutes. The closed-loop circuit generated digital latencies during each trigger, which were described by their minimum (quantile 0), 25% quantile (quantile 1), median (quantile 2), 75% quantile (quantile 3), and maximum (quantile 4). Their results are described in the Results section of this Appendix S1 on p. 22.

Study parameters of iART

The automated triggering of the videos was indicated by the rise of the square wave signal in the trigger channel. The patient responses to the videos were scored manually by medical staff attending the test session using “0” for missed response, “1” for incorrect response, and “2” for correct response. The three types of possible responses (rows) were sorted into two columns according to a true-positive (IED-burst) or false-positive (normal EEG) trigger (visually verified in the offline analysis). From the 3x2 contingency table containing the number of correct, incorrect, or missed responses during either normal EEG or IED-bursts, the probabilities of incorrect and missed responses during IED-bursts and normal EEG were calculated. The probabilities of incorrect and missed responses due to IED-bursts were the IED-associated probabilities for incorrect and missed responses adjusted for the percentages of incorrect or missed responses during normal EEG (i.e., subtracted from each other). For the true-positive triggers, the IED-burst duration (ms) and the trigger time (ms) from the beginning of the IED-burst to the rise of the square wave in the trigger channel were measured in the EEG review software.

Statistical Analysis

The statistics of the patient responses were calculated with GraphPad Prism 10 (main text; Table S2, rows 30-32 and respective columns). For each parameter, the mean (SD) and the median with 95% Confidence Interval (95%-CI) were calculated. Most pairs of parameters were tested with the Wilcoxon paired signed-rank test. When 3 or more parameters were tested, the non-parametric Friedman test was used. Correlation was computed between several pairs of parameters. Results were significant at corrected *p* 2-tailed <0.05.

Model classification performance

During the offline analysis of the EEGs and test sessions in the prospective pilot study, the timestamps and the square wave signals in the trigger channel were used to determine if a trigger occurred during an IED-burst (true-positive detection) or during normal EEG (false-positive detection). The criteria for true-positive detections and for false-negative detections, i.e., IED-bursts that were missed by the algorithm, correspond to the IED-bursts as described in the “IED definition” part of the main text’s Methods section. False-positive detections were, for example, triggers by eye or motion artifact. When the triggers were caused by EEG changes that narrowly missed the IED definition, the assignment to true-positive or false-positive was based on the observer's weighting. The number of true-negative classifications (i.e., no trigger during normal EEG) had to be calculated, because the command window of the IED-detection model did not count the total number of classifications at the time when the study was made. To obtain the total number of classifications made by the model, the duration of a test session in minutes was multiplied by 60 and 5 (latency of ≈ 0.2 s to process 200 microvolt values (i.e., one window) of the ongoing EEG). The number of true-negative classifications was the calculated as the total number of classifications minus the visually validated number of true-positive (TP), false-positive (FP), and false-negative (FN) classifications. Sensitivity was TP/(TP+FN); specificity was TN/(TN+FP). The false-positive rate was the number of false-positive classifications divided by the duration of the test session in minutes.

**Results**

**Optimization of IED-detection model performance**

The metrics we used to measure the classification performance of our model are shown in bold and are explained below. The **sensitivity** is the ratio of $\frac{True pos}{True pos + false neg}$ classifications. The **specificity** is the ratio of $\frac{True neg}{True neg + false pos}$ classifications. The **F1 score** is the harmonic mean of precision (positive predictive value) and sensitivity. The **Matthews correlation coefficient** (MCC) is a measure of the quality of binary (two-class) classification, here classification of IED-bursts and normal EEG, even when the two classes differ greatly in their sample size ("0" ≈ classification no better than chance, ”+1" ≈ perfect classification). The **negative predictive value** (NPV) is the ratio of $\frac{True neg}{True neg + false neg}$ classifications. A receiver operating characteristic (ROC) curve plots the true-positive rate (sensitivity, $\frac{True pos}{True pos + false neg}$) against the false-positive rate (or 1-sensitivity, $\frac{False pos}{False pos + true neg}$). It thereby illustrates the (theoretical) performance of a (binary) classifier at varying values of the false-positive rate. **ROC-AUC** is the Area Under the (ROC-) Curve. The higher ROC-AUC is the better the model can distinguish IED-bursts from normal EEG. ROC-AUC is sensitive to class imbalance, i.e., it can be biased by an unequal number of samples in the two classes. The reason is that the ratios representing the classification of the positive and negative class are plotted against each other. If, for example, the true negative class (i.e., in our case data points of normal EEG) is much larger than the true positive class, then the false-positive rate will be biased by the number of samples in the true negative class (see equation above, i.e., the false-positive value in the nominator of one of the ratios (x-axis) defining the ROC curve will (artificially) appear much smaller). **PR-AUC** is the area under the precision-recall curve and, like ROC-AUC, is a quantitative measure of classification strength. PR-AUC is less sensitive to class imbalance. The reason for this is that the precision-recall curve represents the ratio of the classification of the true class (sensitivity, $\frac{True pos}{True pos + false neg}$) to a ratio composed only of the positive classifications of both classes, here the number of true positive IED-burst classifications and false positive classifications of the normal EEG ($\frac{True pos}{True pos + false pos}$).^58^ That is, the number of true negative samples has no effect on PR-AUC.

The baseline performance of the IED-detection model was moderate when it was trained with data points of normal EEG and IED-bursts in a 1:1 ratio (Table S3, row 2). Since EEGs of PWE usually contain many more data points of normal EEG than IED-bursts, the first approach was to tackle the class imbalance between normal EEG and IED-bursts. We modified ResNet by exchanging its default global CE loss function with the focal balanced CE loss function. Exchanging the loss function improved classification performance on average by 10% for all metrics except for NPV and ROC-AUC (Table S3, row 3). We then trained the model with varied ratios of normal EEG and IED-burst data points. Normal EEG and IED-bursts in a 5:1 ratio improved most metrics by an additional 10%, except for NPV (Table S3, row 4). The next approach was to pre-train ResNet34 to achieve a lower local minimum in the loss function, from where the IED-detection model could better generalize when presented with new data. We first applied transfer learning with domain adaptation by retraining ResNet34, which was pre-trained with naturalistic images, with EEG data for 100 epochs. This improved most performance metrics by an additional 1-6% (Table S3, row 5). Preliminary experiments showed that classification performance improved when all layers of ResNet34, which were pre-trained on all EEGs from our dataset for 100 epochs, were retrained (fine-tuned) for 5 and 10 epochs with segments of 44 EEGs from the Bern dataset that had a good signal-to-noise ratio (Figure S1). We fine-tuned ResNet34, which had been pre-trained with naturalistic images and trained with all EEGs from our dataset for 100 epochs, with the first 15% from each of the 44 EEGs of the Bern dataset recorded during the flash test for 10 epochs (Table S1). Specificity, F1-score, MCC, and PR-AUC improved by an additional 5-11 % (Table S3, row 6). **This version** (arrowhead) **was used as the fully optimized IED-detection model in the prospective pilot study**. Changing the depth and type of the neural network did not consistently improve the metrics, such as replacing pre-trained ResNet34 with pre-trained ResNet50 (data not shown). Using a simple CNN with 10 layers instead of ResNet34 worsened multiple metrics considerably (Table S3, row 7 vs. row 2). This result justifies using more complex models including the model ultimately used (Table S3, row 6).

**Digital latencies of the closed loop**

The digital latencies of the closed loop, which were only recorded in the car test for RT-measurements, because they required millisecond resolution, had the following median quantiles per person when calculated from the means of all test sessions: 40 ms (minimum), 59 ms (25% quantile), 68 ms (50% quantile or median), 78 ms (75% quantile), 120 ms (maximum) and differed significantly among each other (Table S2, column AE; *p < 0.02*, Friedman test). Note the asymmetry of the median to the 25% quantile (68-59 = 9 ms) and minimum (68-40 = 28 ms) when compared to the 75% quantile (78-68 = 10 ms) and the maximum (120-68 = 52 ms). This observation justified the correction of the digital latency using the photo-voltaic sensor and measuring the effective RTs (Appendix S1, p.5).

**Prospective, non-randomized pilot study**

**Association of electrophysiological IED-burst characteristics with their clinical correlates**

In the car test, the median duration of the IED-bursts without crash was 1238 ms (95%-CI 880-1709), which was significantly shorter than the median duration of IED-bursts with crash, which was 1640 ms (95%-CI 1307-3194) (*p = 0.02*, Wilcoxon test; Table S2, columns V and X). In iART, the median duration of the IED-bursts with correct, incorrect, or missing response was 1459 ms (95%-CI 806-4350), 3347 ms (95%-CI 1832-5234), or 3775 ms (95%-CI 843-10694), respectively Table S2, columns AT, AV, and AX). There were not enough paired data for the comparison using the non-parametric Friedman test. The median trigger time per person was 844.5 ms (95%-CI 758.4-965.5) in the car test and 926.0 ms (95%-CI 585.0-1160.0) in iART. These times did not differ (*p = 0.74*, Wilcoxon test, Table S2, columns AD vs. BE). IED-bursts were with 2.2/min more prevalent in the car test (Table S2, column S, row 29) than with 1.0/min in iART (Table S2, column S, row 29; *p < 0.01*, Wilcoxon test; Table S2, columns S vs. AP, row 31).

**Anatomical-functional specificity of IED-bursts using one detection-channel for technical reasons**

Incorrect or missed responses in neuropsychological tasks are binary events, just like crashes in the driving game (in contrast to the continuous parameter reaction time that has the highest sensitivity to detect IED-burst effects). The probabilities of crashes in the car test and of incorrect or missed responses in iART are in this chapter only from now on referred to as error rates. **Assuming that the underlying functional network is always equally sensitive to the effects of IED-bursts**, the error rates for iART and the car test should be similar from a probabilistic point of view and therefore should not differ substantially at low or high anatomical-functional specificity of the IED-bursts. Table 1 in Appendix S1 (below) lists all patients (from Table S2) for whom error rates were recorded in both iART and the car test.

| Patient No./handiness (L/R), IED detection channel | Epilepsy type/syndrome | IED type in car test & iART | I–D - norm EEG crash-rate [%] | I–D - norm EEG incorrect response rate [%] | I–D - norm EEG missed response rate [%] |
| --- | --- | --- | --- | --- | --- |
| 1 | fE-sE (cystic lesion temporal left) |  |  |  |  |
| 2, R, T4-T6 | GGE | gen atyp | 2.0 | 1.9 | 0 |
| 3 | fE-sE (after DNET resection temporal left) |  |  |  |  |
| 4 | TLE left |  |  |  |  |
| 5 | fE-uE | Focal, gen atyp | 0 | 0 | 0 |
| 6, R, F4-C4 | CAE | gen typ | 3.9 | 9.9 | 25 |
| 7, L, F4-C4 | fE-uE | gen atyp, gen typ | 27.5 | 8.3 | 8.3 |
| 8 | JAE |  |  |  |  |
| 9 | fE-uE | Focal, gen atyp | 0.5 | 0 | 0 |
| 10 | fE-uE | focal | 0 | 0 | 0 |
| 11, L, F4-C4 | Multifocal E-uE | Mostly focal, few gen atyp | 0.1 | -2.8 | 1.7 |
| 12 | DEE: Lennox-Gastaut syndrome, double cortex syndrome |  |  |  |  |
| 13 | fE-uE | Mainly focal | 0.5 | 0 | 0 |
| 14, R, F7-FT9 | TLE left | Focal, gen atyp | 1.3 | 0 | 3.4 |
| 15 | fE-uE | Gen atyp | 0 | 0 | 0 |
| 16 | fE-uE |  |  |  |  |
| 17 | IGE |  |  |  |  |
| 18 | SeLECTS |  |  |  |  |
| 19 | Suspicion of occipital lobe epilepsy |  |  |  |  |
| 20, R, F4-C4 | Atypical absence epilepsy | Focal, gen atyp, gen typ | 5.7 | 7.1 | 0 |

**Appendix S1: Table 1**

At first glance, Table 1 of Appendix S1 does not show any major discrepancies in the error rates between iART and the car test, i.e., low error rates in iART corresponded in principle to low error rates in the car test and vice versa. Only patients 6 and 7 seemed to be different.

First, the relation between handiness and choice of the IED-detection channel, i.e., the anatomical-functional specificity of the IED-bursts is discussed for each patient. Afterwards, the error rates in iART and the car test are re-evaluated after correction for the effects of the electrophysiological IED-burst characteristics, since, for example, longer IED-burst duration correlates with higher error rates, as previously shown.^e.g.,2^

Patient 2 had epilepsy with generalized tonic-clonic seizures and absences with eyelid myoclonias (possible Jeavons Syndrome) and was right-handed. The EEG during testing showed generalized atypical (not-well organized) IED-bursts. The IED-detection channel was T4-T6 (Table 2 of Appendix S1). Since the IED-bursts were generalized, it is unlikely that the rate of missed responses in iART would have been higher if a left-hemispheric channel was picked for IED-burst detection. Thus, the **anatomical-functional specificity of IED-bursts** was **preserved.**

Patient 6 had childhood absence epilepsy was right-handed and the generalized s/w discharges were symmetrically distributed across both hemispheres. Channel F4-C4 was used for IED-burst detection (Table 2 of Appendix S1). Because of the symmetry of the discharges, it is unlikely that the choice of the IED-detection channel mattered. Therefore, iART results likely reflect the true prevalence of error probabilities in neuropsychological tasks. The **anatomical-functional specificity of IED-bursts** was **preserved.**

Patient 7 had focal epilepsy of unknown etiology; a rapidly generalizing frontal lobe epilepsy was assumed. This patient was left-handed and channel F4-C4 was picked for detecting IED-bursts. The IED-bursts were focal and generalized. The generalized IED-bursts had both well organized (typical) and not-well organized (atypical) morphology. With IED-detection channel F4-C4, the **anatomical-functional specificity** between IED-bursts and the cortical areas for simple motor tasks and language were **preserved**. It can be assumed that the true prevalence of error probabilities in neuropsychological tasks was measured.

Patient 11 was diagnosed with multifocal epilepsy of unknown origin and was left-handed (i.e., this patient’s language zone was presumedly more right-than left-hemispheric). Channel F4-C4 was used for IED-burst detection (Table 2 of Appendix S1). Patient 11 had focal IED-bursts in the central/midline channels more on the right than on the left hemisphere with rare generalization. During testing, no IED-bursts were seen in any other location. The **anatomical-functional specificity of IED-bursts** was also **preserved** in this patient, and it is likely that the iART results reflect the true prevalence of error probabilities in neuropsychological tasks.

Patient 14 had left temporal lobe epilepsy and had undergone left temporal pole resection due to an encephalocele. Patient 14 was right-handed. Channel F7-FT9 was picked for detecting IED-bursts, which probably also contained a breach effect. Approximately 10% of the detected IED-bursts were truly focal while about 90% were generalized atypical, thus spreading also to the right hemisphere. In left-handers, the language zone is supposed to be in both hemispheres, with a predominance on the right side. It can be assumed that the error rates in neuropsychological tasks would not have been considerably higher if a right-hemispheric channel had been picked. It is therefore likely, that the **anatomical-functional specificity** of IED-bursts was **preserved**.

Patient 20 had atypical absence epilepsy and was right-handed. Channel F4-C4 was picked for IED-burst detection. IED-bursts could be focal (incompletely generalized, generalized atypical (not well organized), and generalized typical (well organized). 121 IED-bursts were detected during both tests together. 19 of 121 IED-bursts were focal (15%). The anatomical-functional specificity for these 15% of focal IED-bursts was suboptimal. The remaining 85% IED-bursts were generalized. It can be assumed that for 85% of the responses where it did not matter whether the detection channel was on the (correct) contralateral side of the language zone. It is therefore likely that the **measured error rates** were **not significantly lower than their real percentages**.

This detailed discussion of each patient regarding the localization of the IED-bursts and the choice of IED-detection channel shows that the anatomical-functional specificity IED-bursts was rather preserved in this pilot study. We believe that error rates in neuropsychological tasks could be higher with faster IED-detection and video triggering. Median duration of IED-bursts associated with incorrect and missed responses was 3555ms (Table S2, column AV, row 34). The median trigger time was 926 ms (Table S2, column BE), and the videos were on average 2-3 seconds long, meaning that the effect of the IED-bursts was rather measured at their end than in their middle.

Since the electrical properties of IED-bursts influence their clinical correlates,^e.g.,2-5^ the morphology and duration of IED-bursts and the width of their epileptogenic field are now also considered. IED-bursts have a spontaneously variable appearance (in the EEG), and their prevalence fluctuates during the day. As the car test and iART were performed in quick succession, diurnal rhythms should have less influence on the prevalence and appearance of IED-bursts. Therefore, from a statistical point of view, their spontaneous prevalence, morphology, width of the epileptogenic field (focal or generalized), and duration in both tests should be comparable, as should, in theory, their clinical impact. The differences in the electrical IED-burst properties between iART and the car test should thus mainly be due to the interaction of an epileptic brain with the variable complexity of the test tasks and the localization of the involved cerebral network. While visually the width of the epileptic field and the morphology, as well as statistically the IED-burst duration (Wilcoxon test: IED duration_crashes_ vs. IED duration_incorrect responses_ *p* = 0.38; IED duration_crashes_ vs. IED duration_missed responses_ *p* > 0.99) did not differ between the car test and iART, higher attention in iART significantly suppressed IED-burst prevalence compared to the car test (*p* = 0.02, Wilcoxon test, Table S2, column S vs. AP). Since we cannot correct for IED-burst prevalence, we can at least correct for the influence of IED-burst duration on the error rates of individual test sessions. The duration of IED-bursts associated with crashes, incorrect responses, or missed responses could not be pairwise compared (Friedman test) because of too few paired values in the three groups. If the duration of IED-bursts associated with crashes was compared with the duration of IED-bursts associated with incorrect responses, or with IED-bursts associated with missed responses, no significant difference emerged (*p > 0.8*, Wilcoxon test, Table S2, columns X, AV, and AX). Table 2 of Appendix S1 shows the error rates and the measured IED durations on a session level.

| Patient No. | Crash-probability IED-norm EEG | Mean duration of IEDs w/crash | IED-norm incorrect response probability | Mean duration of IEDs w/incorrect response | IED-norm missed response probability | Mean duration of IEDs w/missed response |
| --- | --- | --- | --- | --- | --- | --- |
| 2 | 2.0% | 3194ms | 1.9% | 1832ms |  |  |
| 6 | 3.9% | 12367ms | 9.9% | 5234ms | 25% | 10694ms |
| 7 | 27.5% | 3313ms | 8.3% | 4790ms | 8.3% | 5230ms |
| 11 | 0.1% | 1061ms | -2.8% | 0ms, no IEDs | 1.7% | 843ms |
| 14 | 1.3% | 1339ms |  |  | 3.4% | 2320ms |
| 20 | 5.7% | 1505ms | 7.1% | 1904ms |  |  |

**Appendix S1: Table 2**

Patient 2 had both lower rates of incorrect responses in iART and a shorter duration of IED-bursts compared to the crash rate and the IED-burst duration in the car test. A shorter duration of IED-bursts may reflect the increased attention that is required to perform iART in comparison to the car test.^2,59^ If the rate of incorrect responses in iART is calculated in such a way that the mean IED-burst duration in iART is the same as the mean IED-burst duration in the car test, then the rate of incorrect responses to neuropsychological tasks was with 3.5% **higher** than the crash rate of 2% in the car test.

Patient 6: If the rate of incorrect responses and missed responses in iART is calculated in such a way that the duration of IED-bursts associated with incorrect and missed responses is the same as the duration of IED-bursts associated with crashes in the car test, then the rate of incorrect and missed responses to neuropsychological tasks was with 23.4% and 28.9%, respectively, much **higher** than the crash rate of 3.9% in the car test.

Patient 7: If the rate of incorrect responses and missed responses in iART is calculated in such a way that the duration of IED-bursts associated with incorrect and missed responses is the same as the duration of IED-bursts associated with crashes in the car test, then the rate of incorrect and missed responses to neuropsychological tasks was with 5.7% and 5.3%, respectively, **much lower** than the crash rate of 27.5% in the car test.

Patient 11: If the rate of missed responses in iART is calculated in such a way that the IED-burst duration in iART is the same as the mean IED-burst duration in the car test, then the rate of missed responses to neuropsychological tasks was with 2.1% **higher** than the crash rate of 0.1% in the car test.

Patient 14: If the rate of missed responses in iART is calculated in such a way that the IED-burst duration in iART is the same as the IED-burst duration in the car test, then the rate of missed responses to neuropsychological tasks was with 2.1% **higher** than the crash rate of 0.1% in the car test.

Patient 20: If the rate of incorrect responses in iART is calculated in such a way that the IED-burst duration in iART is the same as the IED-burst duration in the car test, then the rate of incorrect responses to neuropsychological tasks was with 5.6% **approximately the same** as the crash rate of 5.7% in the car test.

It has been shown before that IED-burst effects on neuropsychological tasks including language can be stronger than on simple motor tasks. It was also shown that speech with low information content was less affected than speech with high information content.^60^ Our data seem to support this observation, except for patient 7. While the greater impact of IED-bursts on cognitive functions than on simple motor tasks has been explained by a cerebral capacity theory, i.e., the brain’s limited capacity to process higher entropy information,^60^ with this capacity being additionally impaired by IED-bursts in a probabilistic and unpredictable way,^61^ this theory should also be applicable to patient 7. The only current explanation for the differing findings in **patient 7** is that the underlying **functional networks are not equally sensitive to the effects of IED-bursts**. In other words, there may be an additional susceptibility of the cerebral network to long-standing epilepsy, mediated, for example, by a structural change such as a regionally increased loss of inhibition, even though patient 7 is MR-negative for structural epileptogenic lesions. More information must be collected about cerebral information processing and local network susceptibilities to IED-bursts.

**Example of a Research Report that was prepared for each patient and sent to the referring physician**

**Research report for Patient No. 20 (Table S2) using the Digital Response Test in Epilepsy on 10/18/2024**

**Diagnosis**

Atypical Absence epilepsy

Seizure semiology/EEG: mostly generalized discharges, either sharp theta, or atypical and typical spike-wave morphology.

Antiseizure medication: none, ETX stopped in 7/2024.

Cerebral MRI: not yet performed (scheduled 12/2024).

**Test parameters**

Channel in longitudinal bipolar montage for IED-burst detection (choice based on prominent EEG change & relatively few artifacts): F4-C4

**Car test**

Test duration: 35 min

Recorded results

337 obstacles triggered during the test session: 244 obstacles during normal EEG, 93 obstacles during IED-bursts.

- IED-burst frequency/min: 2.7
- Measured mean (SD) reaction time during normal EEG: 488.1 ± 156.6 ms
- Measured mean (SD) reaction time during IED-bursts: 593.2 ± 131.8 ms
- *Measured IED-associated reaction time prolongation: 105.1 ms*
- Measured frequency of missed reactions during normal EEG (due to inattention): 0.8%
- Measured frequency of missed reactions during IED-bursts: 6.5%
- *Measured IED-associated frequency of missed reactions: 5.7%*
- *Calculated cumulative risk for an IED-associated missed reaction in the future: 27.4%*

Based on a non-linear relationship between IED-associated reaction time prolongation and accumulated probability for a missed reaction [1].

**interictal Automated Responsiveness Test (iART)**

Test duration: 13 min

69 videos were triggered during the test session: 28 videos during IED-bursts, 41 videos during normal EEG, 10 videos were for memorization of time (“remember the time”), 59 videos were with patient’s responses.

- IED-burst frequency/min: 2.2
- Measured frequency of incorrect responses during normal EEG (due to inattention): 0.0%
- Measured frequency of incorrect responses during IED-bursts: 7.1%
- *Measured-IED associated frequency of incorrect responses: 7.1%*
- Measured frequency of missed responses during normal EEG (due to inattention): 0.0%
- Measured frequency of missed responses during IED-bursts: 0.0%
- *Measured-IED associated frequency of missed responses: 0.0%*

**Interpretation**

The EEG changes of patient No. 20 have clinical correlates. They prolong reaction times on average by 100ms. In the car test that measures reactivity, they haven an IED-associated frequency for missed responses of about 5%. In iART that measures neuropsychological functions, the EEG changes have an IED-associated frequency for an incorrect response of 7.1%. We consider these EEG changes to be clinically relevant [1].

Question is, antiseizure treatment is restarted, and the patient is retested, to see if the IED-bursts/their effects are suppressed, and if patient benefits in daily life without an increase in side effects.

Reference

1. Krestel H, et al. Predictive Power of Interictal Epileptiform Discharges in Fitness-to-Drive Evaluation. Neurology. 2023;101:e866-e878. doi: 10.1212/WNL.0000000000207531

**Discussion**

**Different effects on behavior and cognition due to IED-bursts with similar appearances**

We analyzed data from 6 patients regarding the different effect of IED-bursts on cognitive function including language and a simple motor task. The anatomical-functional specificity of IED-bursts,^61^ was largely maintained in these patients.

In our study, the data suggest that electrophysiological IED-burst characteristics did not necessarily explain the differences in IED-burst effects on tasks of varying complexity. Cognitive tasks that required increased attention tended to shorten the duration of IED-bursts. After correcting for this effect, i.e., when error rates were calculated based on the same mean IED burst duration in both the car test and iART, cognitive tasks with higher information content (i.e., the iART tasks) were more affected by the IED-burst effects than the response in the car test, which is a cognitive task with lower information content.

The focal or generalized extent of the IED-bursts and their morphology, which reflects the strength and the propagation of the excitatory process, were from a statistical point of view comparable between the car test and iART, because both tests were performed in quick succession on the same day (depending on the question of the referring physician, but typically the car test was performed first). In 5 of 6 patients, cognitive tasks were more affected by IED-bursts than the simple motor task in the car test. One theory suggested that this was due to the information content of the stimulus (i.e., the entropy of the stimulus) and the brain's limited ability to process information, and that this capacity was further impaired by epileptic discharges.^60^ Since iART tasks have a higher information content (and thus higher entropy) than the car test, the different error rates would therefore be explained by the physiologically limited properties of the brain in conjunction with the electrical (and metabolic) interference factors caused by the IED-bursts. This theory does not explain the results in patient 7, in whom the processing of stimuli with high information content was less impaired by IED-bursts than the processing of stimuli with low information content. It must be assumed that, in addition to the entropy of the test tasks and the brain's processing capacity, there must be further regionally varying changes, e.g., hard-wired (but not necessarily MRI-positive) due to the type and duration of epilepsy. A regional network change could be visualized, e.g., by diffusion tensor imaging (DTI) or magnetoencephalography (MEG). Fine histological changes as a consequence of epilepsy, which lead, e.g., to increased inhibition loss in the language network compared to the premotor network, cannot yet be visualized with currently available imaging techniques.

**Considerations on the IED-detection model**

Our study aimed at detecting IED-bursts ultra-fast with high sensitivity and specificity and combining detection with stimulus triggering to permit studying maximal IED-burst effects on human cognition and behavior. Since we assumed that single interictal spikes and IED-bursts cannot be predicted and thus are a different stochastic entity than seizures, we analyzed windows of normal EEG and IED-bursts (from a continuous EEG recording) under the assumption that they fulfill the Markov property, that is, that their future occurrence is independent of the past EEG. As an EEG is a time series of microvolt values, IED-burst detection can be modeled as a first-order Markov chain, that is, a discrete-time stochastic process that satisfies the Markov property. The EEG is used together with the sliding window technique to continuously transform the EEG recording window-by-window and immediately and continuously classify these windows. The use of first-order Markov chains and a sliding window technique, as described herein only for segmenting and analyzing an ongoing EEG recording, instead of having a second window follow the current window to learn the EEG characteristics over time or using one dimension of a recurrent neural network or CNN to share parameters over time, is very well-suited for IED-burst detection. Therefore, our model did not learn the temporal course of the EEG.

Markov Transition Fields (MTF) build on an interesting development which is the transformation of time series into transition networks and characterization of the relationships of connections between the nodes of the network.^20,53^ Transition networks discretize a time series into quantiles and preserve the temporal order of observations. The quantiles are weighed and formed the nodes of a network. One method used a Markov transition matrix to map a time series into a network and calculate the transition probabilities between the network nodes. This function was surjective, that is, the original time series could be reconstructed from the subsequently visualized network.^20^ Transformation and inverse operation were robust since they were repeated and still much of the information of the original times series was retained. The MTF-technology takes the Markov transition matrix a step further by creating a (second) Markov Transition Field matrix from the Markov transition matrix and visualizes adjacent signals in a time series using the transition probabilities of the Markov transition matrix. When the MTF technology is applied to EEG, some of the EEG information is lost (such as the absolute amplitudes due to the normalization of the transition probabilities), but most of the information of the original EEG is retained. In theory, the MTF-technology should also be a surjective function, which would open up the possibility of creating and storing image databases of EEG.

**References in Appendix S1**(using the order of the main text)

1. Krestel H, Schreier DR, Sakiri E, et al. Predictive Power of Interictal Epileptiform Discharges in Fitness-to-Drive Evaluation. *Neurology*. 2023 Aug 29;101(9):e866-e878. doi: 10.1212/WNL.0000000000207531
2. Krestel H, Rackauskaite J, Khoueiry M, et al. The interictal automated responsiveness test (iART) analyzes transient cognitive impairment in an international manner. Conference proceeding European Epilepsy Congress; 2022 [https://onlinelibrary.wiley.com/doi/10.1111/epi.17388; https://www.researchgate.net/publication/362263877_The_interictal_Automated_Responsiveness_Test_iART_analyzes_transient_cognitive_impairment_in_an_international_manner#fullTextFileContent](https://eec2022.abstractserver.com/program/#/details/presentations/1120); Last accessed in January 2025

16. Lin T-Y, Goyal P, Girshick R, He K, Dollár P. Focal loss for dense object

detection. *arXiv*:1708.02002 pages 2980-2988, 2018;

doi.org/10.48550/arXiv.1708.02002

1. Iakubovskii P, Camargo C, Anand G, Hoho2b Ch. Classification models zoo - keras (and tensorflow keras), <https://github.com/qubvel/classification_models>; Accessed January 2025
2. Campanharo AS, Sirer MI, Malmgren RD, Ramos FM, Amaral LA. Duality between time series and networks. *PLoS One*. 2011;6(8):e23378. doi: 10.1371/journal.pone.0023378
3. Systems and methods for analysis of interictal epileptiform discharge (IED) effects on behavior and cognition in people with epilepsy. U.S. patent application PCT/US25/29364; 05/14/2025
4. Touloumes G, Morse E, Chen WC, et al. Human bedside evaluation versus automatic responsiveness testing in epilepsy (ARTiE). *Epilepsia*. 2016 Jan;57(1):e28-32. doi: 10.1111/epi.13262
5. Donner RV, Small M, Donges JF, et al. Recurrrence-based time series analysis by means of complex network methods. *International Journal of Bifurcation and Chaos* 2011;21(4):1019-1046. doi.org/10.1142/S0218127411029021
6. Faouzi J, Janati H. pyts: A Python Package for Time Series Classification. *Journal of Machine Learning Research*, Microtome Publishing 2020, 21, pp.1 – 6, hal-02883389
7. He K, Zhang X,Ren S, Sun J. Deep residual learning for image recognition. *arXiv*:1512.03385; doi.org/10.48550/arXiv.1512.03385
8. Deng J, Dong W, Socher R, Li L-J, Li K, L Fei-Fei L. ImageNet: A large-scale hierarchical image database. *IEEE Conference on Computer Vision and Pattern Recognition*. 2009:248-255; doi: 10.1109/CVPR.2009.5206848
9. Desmos Graphing Calculator. Available at: <https://www.desmos.com/calculator/hq6sxa0c8g?lang=de>. Accessed April, 2025
10. Jesse Davis and Mark Goadrich. 2006. The relationship between Precision-Recall and ROC curves. In Proceedings of the 23rd international conference on Machine learning (ICML '06). Association for Computing Machinery, New York, NY, USA, 233–240. doi.org/10.1145/1143844.1143874
11. Kasteleijn-Nolst Trenité DG, Vermeiren R. The impact of subclinical epileptiform discharges on complex tasks and cognition: relevance for aircrew and air traffic controllers. Epilepsy Behav. 2005 Feb;6(1):31-4. doi: 10.1016/j.yebeh.2004.10.005
12. Tizard B, and Margerison JH. Psychological functions during wave-spike discharge. British Journal of Social & Clinical Psychology. 1963 3(1):6–15. https://doi.org/10.1111/j.2044-8260.1964.tb00397.x
13. Shewmon DA, Erwin RJ. The effect of focal interictal spikes on perception and reaction time. II. Neuroanatomic specificity. Electroencephalogr Clin Neurophysiol. 1988 Apr;69(4):338-52. doi: 10.1016/0013-4694(88)90005-3
